# Supplementary material for: Uptake of iron and folic acid supplements among pregnant women in Dar es Salaam, Tanzania
Source: PLOS Glob Public Health. 2025 Oct 6;5(10):e0005278. doi: 10.1371/journal.pgph.0005278 (PMC12500133; doi:10.1371/journal.pgph.0005278)
Supplement: Attachment — Submitted filename: Eunice review-PGPH-D-25-00011_R1 (1).pdf [file pgph.0005278.s002.pdf]

PLOS Global Public Health

Iron and folic acid supplements uptake among pregnant women in Dar es Salaam, Tanzania

--Manuscript Draft--

**Manuscript Number:**	PGPH-D-25-00011R1
**Article Type:**	Research Article
**Full Title:**	Iron and folic acid supplements uptake among pregnant women in Dar es Salaam, Tanzania
**Short Title:**	Iron and Folic acid supplementation in Pregnancy
**Corresponding Author:**	Belinda J Njiro, M.D<br>Muhimbili University of Health and Allied Sciences<br>Dar es Salaam, TANZANIA, UNITED REPUBLIC OF
**Order of Authors:**	Careen E. Koka
	Saida Bakari
	Belinda J Njiro, M.D., MSc
	Bruno F. Sunguya, M.D., MSc, Ph.D.
**Keywords:**	Iron; folic acid; micronutrient supplementation; Pregnant women; Anemia; Anemia in Pregnancy; Tanzania
**Abstract:**	Background<br><br>Anemia in pregnancy is a public health concern globally with the biggest brunt felt in low and middle-income countries (LMICs). Tanzania is no exception. Iron and folic acid (IFA) intake is a proven intervention recommended to address the deficiencies behind anemia in pregnancy. Despite interventions in Tanzania, IFA uptake has remained low owing to various reasons not well documented.<br><br>Materials and Methods<br><br>A quantitative cross-sectional study was conducted to assess levels and determinants of IFA uptake during pregnancy in Dar es Salaam, Tanzania. Data was collected among 428 post-delivery women in postnatal wards of Temeke, Mwananyamala, and Amana regional referral hospitals. Descriptive analyses were conducted to determine levels and characteristics of IFA uptake, whereas bivariate and multivariate analyses were conducted to examine determinants of uptake using SPSS version 23.<br><br>Result<br><br>About one in three pregnant women (31.8%) who attended antenatal care (ANC) at referral hospitals had an appropriate IFA uptake. Adequate IFA uptake was 74% lower among primiparous women compared to multiparous women (aOR=0.26; 95% CI: 0.10 – 0.67). Women who reported five or more ANC visits (aOR=11.7; 95% CI: 1.30 – 63.66) and women from high wealth index families index (aOR=11.68 95% CI 2.91 – 89.57) were 12 times more likely to have adequate IFAS uptake than those with fewer visits respectively. Living in a food-secure household was also associated with higher IFAS uptake by nine times compared to women whose households had severe food insecurity (aOR=9.21 95% CI 1.82 – 10.33).<br><br>Conclusion<br><br>Only 31.8% of pregnant women attending regional referral hospitals in Dar es Salaam had appropriate IFA uptake to prevent anaemia during pregnancy. Such unprecedented low uptake needs to be addressed through targeted interventions among pregnant women with higher parity, fewer ANC attendances, challenging family support, and coming from households with economic hardship and food insecurity.
**Additional Information:**	

Powered by Editorial Manager® and ProduXion Manager® from Aries Systems Corporation

Question	Response
**Financial Disclosure**<br><br>Enter a financial disclosure statement that describes the sources of funding for the work included in this submission and the role the funder(s) played. This includes grants and any commercial funding of the work or authors.<br><br>This statement will be typeset if the manuscript is accepted for publication.<br><br>*Please review the submission guidelines and the instructions link below for detailed requirements and guidance.*	The authors received no specific funding for this work
**Competing Interests**<br><br>On behalf of all authors, disclose any competing interests that could be perceived to bias this work.<br><br>This statement will be typeset if the manuscript is accepted for publication.<br><br>*Please review the instructions link below and PLOS Global Public Health's competing interests policy to determine what information must be disclosed at submission.*	The authors declare no competing interests.
**Data Availability**<br><br>Before publication, Authors are required to make fully available and without restriction all data underlying their findings. Please see our PLOS Data Policy page for detailed information on this policy.<br><br>A **Data Availability Statement**, detailing where the data can be accessed, is	There are legal restrictions to sharing the datasets; we do not have approval to share data in public domains. However, data requests from individuals or entities shall be honored upon fulfilling the national regulatory requirements including data transfer agreement (DTA) as advised by the National Research and Ethics Committee (NIMR) of Tanzania. The datasets used and/or analyzed during the current study can be requested by contacting the Muhimbili University of Health and Allied Sciences (MUHAS) Directorate of Research and Publication at drp@muhas.ac.tz

Powered by Editorial Manager® and ProduXion Manager® from Aries Systems Corporation

required at first submission. Insert your Data Availability Statement in the box below.<br><br>Please see the data reporting section of our submission guidelines for instructions on what you need to include in your Data Availability Statement.<br><br>This statement will be typeset if the manuscript is accepted for publication.<br><br>*PLOS allows rare exemptions to address legal and ethical concerns. If you have legal or ethical restrictions, please use the box below to detail these in full sentences for the Journal team to consider.*	

Manuscript Click here to access/download;Manuscript;IFA supplements manuscript_revised_24May2025_clean.docx

Iron and folic acid supplements uptake among pregnant women in Dar es Salaam, Tanzania

Careen E. Koka[1], Saida Bakari[1], Belinda J. Njiro[1*], Bruno F. Sunguya[1]

[1]School of Public Health and Social Sciences, Muhimbili University of Health and Allied Sciences, 9 United Nations Road, Dar es Salaam, Tanzania

[*]Corresponding Author: Belinda J. Njiro
Email: belindaj.njiro@gmail.com

Department of Epidemiology and Biostatistics, School of Public Health and Social Sciences, Muhimbili University of Health and Allied Sciences.
Address: 9 United Nations Road, Upanga West, P.O Box 65001, Dar es Salaam, Tanzania
Tel: +255 755 226 469

Authors emails:
CEK: careenkoka@icloud.com
SB: saidahmohamed2014@gmail.com
BJN: belindaj.njiro@gmail.com
BFS: sunguya@gmail.com; bruno.sunguya@muhas.ac.tz

Abstract

Background: Anemia in pregnancy is a public health concern globally with the biggest brunt felt in low and middle-income countries (LMICs). Tanzania is no exception. Iron and folic acid (IFAS) intake is a proven intervention recommended to address the deficiencies behind anemia in pregnancy. Despite interventions in Tanzania, IFAS uptake has remained low owing to various reasons not well documented.

Materials and Methods: A quantitative cross-sectional study was conducted to assess levels and determinants of IFAS uptake during pregnancy in Dar es Salaam, Tanzania. Data was collected among 428 post-delivery women in postnatal wards of Temeke, Mwananyamala, and Amana regional referral hospitals. Descriptive analyses were conducted to determine levels and characteristics of IFAS uptake, whereas bivariate and multivariate analyses were conducted to examine determinants of uptake using SPSS version 23.

Result: About one in three pregnant women (31.8%) who attended antenatal care (ANC) at referral hospitals had an appropriate IFAS uptake. Adequate IFAS uptake was 74% lower among primiparous women compared to multiparous women (aOR=0.26; 95% CI: 0.10 – 0.67). Women who reported five or more ANC visits (aOR=11.7; 95%CI: 1.30 – 63.66) and women from high wealth index families index (aOR=11.68; 95% CI: 2.91 – 89.57) were 12 times more likely to have adequate IFAS uptake than those with fewer visits respectively. Living in a food-secure household was also associated with higher IFAS uptake by nine times compared to women whose households had severe food insecurity (aOR=9.21; 95% CI: 1.82 – 10.33).

Conclusion: Only 31.8% of pregnant women attending regional referral hospitals in Dar es Salaam had appropriate IFAS uptake to prevent anaemia during pregnancy. Such unprecedented low uptake needs to be addressed through targeted interventions among pregnant women with higher parity, fewer ANC attendances, challenging family support, and coming from households with economic hardship and food insecurity.

Keywords: *Iron and folic acid, micronutrient supplementation, pregnant women, anemia, anemia in pregnancy, Tanzania*

Introduction

Anemia in pregnancy is diagnosed as hemoglobin concentration of less than 11.0 g/dl in the first trimester, 10.5g/dl in the second trimester and 11.0g/dl in the third trimester (1). Iron deficiency is the most common cause of Anemia in women of reproductive ages and in pregnancy (1). Iron is a crucial component of hemoglobin, a protein found in red blood cells (RBCs) that is responsible for carrying oxygen throughout the body. Human bodies need folic acid for the synthesis, repair, methylation, and cell division of DNA. Folic acid is also needed to develop healthy RBCs and to prevent anemia, especially during pregnancy and infancy (2).

Nutritional anemia is the most common type of anemia in pregnancy and is a significant public health challenge worldwide, particularly in low- and middle-income countries (LMICs)(3). Anemia affects about1.62 billion people globally, accounting for 24.8% of the world's population (4,5). Pregnant women are the most vulnerable populations in nutritional anemia (6); 56.4 million pregnant women are affected by anemia, and of them 17.2 million are from Africa (1). Iron deficiency anemia, accounts for more than half of all occurrences of anemia in pregnancy, which is one of the major contributing factors to the worldwide burden of disease (7).

Pregnancy-related physiological changes, fetal growth, and development raise the need for iron and folic acid (8). Due to the lower bioavailability of nutrients in pregnant women, the increased demand for essential nutrients cannot be satisfied by diet alone (9). If nutrition is not supplemented with iron and folic acid tablets throughout pregnancy, there is a considerable risk that iron deficiency and folic acid deficiency may manifest (9). The World Health Organization (WHO) recommends iron and folic acid supplementation (IFAS) as one of the interventions for the control of anemia in pregnancy (1,7,10).

The WHO recommends that pregnant women take daily doses of 30 to 60 mg of iron and 0.4 mg of folic acid to prevent maternal anemia (11). Iron and folic acid deficiency and anemia in pregnancy have been linked with maternal morbidity including fatigue, breathing difficulties, higher risk of postpartum depression, blood loss during labor, and infections including puerperal sepsis. Negative consequences for the infants include low birth weight, neural tube defects such as spina bifida and anencephaly, intrauterine growth restriction, impaired fetal growth, preterm birth, and perinatal mortality (11–13). The risk of all types of maternal anemia at term is reduced

by 70% and iron deficiency anemia at term by 57%, if daily iron and folic acid supplements (IFAS) are taken throughout pregnancy (1). The risk of infant mortality can also be decreased by 34% if a pregnant woman takes IFAS for 90 days during pregnancy (1). Moreover, the levels of maternal folic acid may also influence infants' intellectual development (14).

To attain a 50% reduction in anemia among women of reproductive age by 2025, the WHO recommends the provision of IFAS in pregnancy (2). Reduced maternal and child morbidity and mortality are already notable with the use of IFAS (7). However, the uptake has remained low in many regions and countries, including Tanzania. Evidence on the associated factors varies and sometimes lacking in some contexts including Tanzania. This study aimed to fill this evidence gap by documenting Iron and folic acid supplement uptake and associated factors among pregnant women in Dar es Salaam, Tanzania

Methodology

Study design and setting

This hospital-based cross-sectional quantitative study was conducted to determine the factors associated with iron and folic acid supplement uptake among pregnant women in Dar es Salaam, Tanzania. The study was conducted from 1st to 31st of July 2024 at three referral hospitals in Dar es Salaam, namely, Temeke, Mwananyamala and Amana regional referral hospitals located in Temeke, Ilala, and Kinondoni districts respectively.

Study population

Data was collected from post-natal women attending the three health facilities at their respective post-natal wards. Both post-spontaneous vaginal delivery and post-cesarean section women that gave birth were eligible to participate and were involved in the study. All post-natal women aged 15 to 49 years visiting the post-natal wards at Temeke, Amana, and Mwananyamala regional referral hospitals were eligible to participate. ~~We included~~ only women with a prior history of antenatal care visits to any health facility during their most recent pregnancy and who had their antenatal care (ANC) cards for quality checks. The study excluded women who were in severe pain, had health emergencies and those who did not consent to participate in the study.

Sampling and sample size

The hospitals were purposively selected owing to the volume of the clients and therefore sample population. Participants were randomly selected for the study using a simple random sampling technique. The sample size was estimated by using Fisher's formula $N=Z^2 P (1-P) / E^2$, where N is the estimated minimum sample size; Z is the confidence level at 95% (standard value is 1.96); P is the proportion (prevalence of iron and folic acid adherence (50%); and E is the precision at 95% CI = 0.05. The minimum sample that was required for this study was 385 post-natal women. A 10% non-response rate was used to give a total sample size of 428 post-natal women. Participants were selected through a simple random sampling technique using a lottery technique whereby participants were assigned numbers each. Numbers were written on small pieces of paper, of the same size, color and shape. The papers were folded and then put in a box and then mixed. A blindfold selection was made. The process of selecting continued until the required sample size

per respective hospital was reached. About 18 – 24 participants were selected in a day.

Data collection and measurements

Data was collected from respondents via face-to-face interviews by the researcher and two trained research assistants using a semi-structured questionnaire adopted from the 2022 Tanzania Demographic Health Survey (TDHS) and previous studies (15,16) All the questions were prepared in English and translated in Swahili (local language). The interviews lasted for approximately 30 minutes for each respondent; data collection was conducted at the respondents' respective postnatal wards. ~~We collected~~ data on maternal age, education level, employment status, and wealth index. Weighted wealth index was used as a proxy for household economic level. It considered the ownership of home resources, dwelling features, fuel for cooking and lighting, toilet type, water supply, and feeding habits was used to determine economic status. The items were weighted and added together to produce the wealth index. The weighted wealth index score was split similarly into three (terciles) representing high, medium, and low economic levels. Maternal factors assessed included parity, timing of the first ANC, and number of ANC visits. Health facility factors included distance to the health facility and HCP's attitude.

The feeding frequency tool was adopted from previous Tanzanian research projects conducted in Tanzania (17).The WHO suggests a feeding schedule of three meals and at the minimum two snacks in 24 hours for pregnant women (18). The dietary Diversity Scale (DDS) was used to measure dietary diversity adopted from previous studies conducted in Tanzania. ~~We extracted~~ a roster of twelve foods from the 2022 Tanzania Demographic Health Survey (TDHS) (15). Participants were asked to mention the food items they had on the earlier day, and we determined the final score for dietary diversification. Dietary diversity below 4 was considered as low. Household food insecurity was assessed using a nine-item Household Food Insecurity Access Score (HFIAS) tool. On this scale, the options are: 0 ="no," 1 = "infrequently," 2 = "spoken" and 3 = "frequently"." The total score ranges from 0 to 27. The food insecurity magnitude can be classified into four categories: highly insecure (18 – 27), severely insecure (11 – 17), mildly insecure (2 – 10), and secure (0 – 1).

The primary outcome for this study was the IFAS uptake of 90 pills or more. IFAS uptake was self-reported by the study participants as the average number of days that participants took iron

and folic acid supplements during their most recent pregnancy (15). This information was double-checked on the Reproductive and Child Health (RCH) card used to record maternal and child assessments and progress during pregnancy and postpartum periods by the healthcare workers. In Tanzania, all pregnant women receive a single pill containing 200mg of ferrous sulphate and 0.4 mg of folic acid daily (19); this is equivalent to 65 mg of elementary iron and 400 μg of folic acid daily starting at the first antenatal visit as WHO recommends (11). WHO recommends a daily intake of iron and folic acid throughout the pregnancy (11). ~~We defined~~ adequate IFAS uptake as the intake of folic acid and iron tablets for a minimum of 90 days per pregnancy adopted from previous studies in similar settings from Tanzania and Pakistan, and using TDHS methodologies (1,20,21). ~~We analyzed~~ IFAS uptake as a categorical variable categorized as normal uptake (90 or more pills per pregnancy) and low uptake (less than 90 pills per pregnancy). The adequate IFAS uptake in our study ~~is~~ equivalent to at least a third of the WHO recommended daily IFAS intake throughout pregnancy (20,21). .

Data analysis

The collected data was analyzed to identify the factors associated with low IFAS uptake using both descriptive and logistic regression analyses. ~~We employed~~ descriptive analysis to summarize the study population characteristics. We analyzed IFAS uptake as a categorical variable with levels, namely adequate uptake (90 pills or more per pregnancy) and low uptake (less than 90 pills per pregnancy). The magnitude of IFAS uptake was calculated as the percentage of IFAS uptake of 90 pills or moreover the total study population. The Pearson Chi-square test was used to examine the differences in the proportions of IFAS uptake by participants' characteristics. Bivariate and Multivariate logistic regression analyses were computed, and crude and adjusted odds ratios with corresponding 95% confidence intervals were recorded. Variables were included in the final model if having a p-value of <0.2 in the bivariate analysis. All analyses were conducted using IBM SPSS version 23.0. A p-value of <0.05 was considered statistically significant.

Ethical considerations

This study received ethical clearance from the Institutional Review Board of the Muhimbili University of Health and Allied Science (MUHAS IRB); ethical approval number MUHAS-REC-06-2024-2348. We sought the necessary permissions from the district administrative office and

the respective hospitals at Temeke, Mwananyamala, and Amana regional referral hospitals. All participants provided written consent to participate before inclusion in the study. For participants aged less than 18 years, we obtained written assent; informed verbal consent was also obtained from their respective guardians. The information obtained from the participants was used for research purposes alone and are available to authorized personnel only.

Patient and Public Involvement

Patient or the public were not directly involved in the design and conduct of this research. However, as part of our dissemination plan, the final report from our analysis will be shared with the included health facilities to inform them of the status of IFAS uptake and the relevant recommendations.

Results

Participant's characteristics

A total of 428 participants (post-delivery women) were recruited for the study with full informed consent. **Table 1** shows the participants' characteristics stratified by the adequacy of IFAS uptake. Among all participants, only 31.8% (n=136) had adequate IFAS uptake during pregnancy. The highest proportion of adequate IFAS uptake was among participants with university-level education (53.8%, n=28), multiparous women (42.0%, n=58), women who booked their ANC visit in the first trimester (53.5%, n=106), and those that had five or more ANC visits. The proportion of adequate IFAS uptake was lower among women in the low wealth index and those who reported experiencing poor healthcare provider attitudes.

Table 1: Uptake of IFAS by participants' characteristics (N=428)

Variables	Total n (%)	Adequate uptake, n (%)	Inadequate uptake, n (%)	*p-value*
**Age groups**				0.688
16 – 25	188 (43.9)	56 (41.2)	132 (45.2)	
26 – 35	194 (45.3)	62 (45.5)	132 (45.2)	
36 – 45	46 (10.7)	18 (13.2)	28 (9.6)	
**Highest education level**				
Primary level	142 (33.2)	52 (34.7)	90 (33.1)	0.027
Secondary level	228 (53.3)	70 (46.7)	158 (58.1)	
University	52 (12.1)	28 (18.7)	24 (8.8)	
**Parity**				
Primiparity	164 (38.3)	38 (27.9)	126 (43.2)	0.046
Secundiparity	126 (29.4)	40 (29.4)	86(29.5)	
Multiparity	138 (32.2)	58 (42.6)	80(27.4)	
**Time of first booking**				
1st trimester	198 (46.3)	106 (70.7)	92 (33.8)	0.003
2nd& 3rdtrimester	200 (46.7)	44 (28.2)	156(57.4)	
Don't know	30 (7.0)	6 (8.8)	24 (8.8)	
**Number of ANC visits**				
1 – 4	168 (39.3)	24 (17.6)	144 (49.3)	<0.001
5 and above	212 (49.5)	104 (76.5)	108 (37.0)	
Don't know	48 (11.2)	8 (5.9)	40 (13.7)	
**Wealth index**				

High	166 (38.8)	74 (54.4)	92 (31.5)	0.001
Middle	173 (40.4)	55 (40.4)	118 (40.4)	
Low	89 (20.8)	7 (5.1)	82 (28.1)	
**Distance to health facility affecting treatment**				
Yes	158 (36.9)	36 (26.5)	122 (41.8)	0.031
No	270 (63.1)	100 (73.5)	170 (58.2)	
**Healthcare worker's attitude**				
Good	292 (68.2)	106 (77.9)	186 (63.7)	0.037
Fair/bad	136 (31.8)	30 (22.1)	106 (36.3)	
**Household food security**				0.011
Food secure	226 (52.8)	95 (69.9)	131 (44.9)	
Mildly insecure	30 (7.0)	11 (8.1)	19 (6.5)	
Moderately insecure	77 (18.0)	15 (11.0)	62 (21.2)	
Severely insecure	95 (22.2)	15 (11.0)	80 (27.4)	
**Dietary diversity score**				0.159
Below 4	208 (48.6)	55 (40.4)	153 (52.4)	
4 and above	220 (51.4)	81 (59.6)	139 (47.6)	
**Feeding frequency**				0.002
Low	143 (33.4)	22 (16.2)	121 (41.4)	
Good	285 (66.6)	114 (83.8)	171 (58.6)	

Factors associated with adequate iron and folic acid uptake

Table 2 shows the bivariate and multiple logistic regression analysis findings. The odds of adequate IFAS uptake were 74% lower among primiparous women as compared to multiparous women (aOR=0.26; 95% CI: 0.10 – 0.67). Women who reported five or more antenatal visits were 12 times more likely to have adequate IFAS uptake than those who attended 1–4 ANC visits (aOR=11.7; 95% CI: 1.62 – 84.34). Women from high-wealth index families were12 times more likely to report adequate IFAS uptake than women whose families had low wealth index (aOR=11.68; 95% CI: 2.91 – 89.57). Living in a food-secure household was also associated with higher IFAS uptake by nine times compared to women whose households had severe food insecurity (aOR=9.21; 95% CI: 1.82 – 10.33).

Table 2: Bivariate and multivariate analysis for factors associated with IFAS uptake

Variable	Bivariate analysis		Multivariate analysis	
	cOR (95%CI)	*p value*	aOR (95%CI)	*p value*
**Highest education level**				
Primary	0.29 (0.11 – 0.74)	0.010	0.54 (0.16 – 1.91)	0.341
Secondary	0.38 (0.16 – 0.90)	0.029	0.65 (0.20 – 2.11)	0.474
University	Reference		Reference	
**Parity**				
Primiparity	0.42 (0.21 – 0.84)	0.014	0.26 (0.10 – 0.67)	0.005
Secundiparity	0.64 (0.31 – 1.31)	0.223	0.58 (0.23 – 1.47)	0.246
Multiparity	Reference		Reference	
**Time of first booking**				
1st trimester	3.07(0.82 – 11.57)	0.097	0.82 (0.09 – 7.19)	0.857
2nd& 3rdtrimester	1.13 (0.29 – 4.26)	0.861	0.54 (0.06 – 4.95)	0.585
Don't know	Reference		Reference	
**Number of ANC visits**				
1 – 4	0.83 (0.24 – 2.87)	0.772	2.23 (0.29 – 16.86)	0.439
5 and above	4.82 (1.54 – 15.04)	0.007	11.70 (1.30 – 63.66)	0.015
Don't know	Reference		Reference	
**Wealth index**				
High	16.25 (3.0 – 88.9)	0.001	11.68 (2.91 – 89.57)	0.059
Middle	6.96 (1.3 – 36.5)	0.022	6.59 (0.69–62.96)	0.102
Low	Reference		Reference	
**Distance to health facility**				
Yes	1.99 (1.06 – 3.75)	0.032	1.92 (0.82 – 4.51)	0.135
No	Reference		Reference	
**Health care workers attitude**				
Good	0.50 (0.26 – 0.97)	0.039	0.62 (0.25 – 1.56)	0.313
Fair/bad	Reference		Reference	
**Household food security**				
Food secure	6.50 (1.73 – 24.39)	0.006	9.21 (1.82 – 10.33)	0.047
Mild food insecurity	4.50 (0.54 – 37.38)	0.164	8.96 (0.16 – 13.13)	0.234
Moderate food insecurity	1.33 (0.26 – 6.83)	0.730	4.15 (0.47 – 6.24)	0.820
Severe food insecurity	Reference		Reference	
**Dietary diversity score**				
<4 (low)	1.96 (0.77 – 4.99)	0.161	0.73 (0.21 – 2.51)	0.617
4 and above (good)	Reference		Reference	
**Food frequency**				
Low	5.47 (1.83 –16.39)	0.002	1.75 (0.31 – 9.82)	0.526

Good	Reference	Reference

aOR: adjusted odds ratio, cOR: crude odds ratio, ANC: antenatal care

Discussion

Less than one in three women had adequate of iron and folic acid supplementation during pregnancy in Dar es Salaam, Tanzania. Inadequate IFAS uptake in the context of the high burden of anemia among pregnant women calls for immediate and sustained efforts to address the burden of anemia in pregnancy. In Tanzania, similar results were documented in the 2022 TDHS, where the IFAS uptake for 90 days and more was reported at 40.8% (16). ~~Our~~ IFAS uptake findings are also not very different from evidence from a neighboring country, Kenya (22). The current findings show an improvement compared to a previous study conducted in Moshi Tanzania where 16.1% of women reported taking folic acid and iron supplements in 2009 (23). This study, however, reported on any use of IFA supplements and did not report on the adequacy of intake. The slight improvement reported could be attributed to improved ANC attendance, access to nutritional counseling at the antenatal clinics, increased number of health facilities and trained health workers, and campaigns on facility-based child deliveries by the government and implementing partners. It is worth noting that the adequacy of IFAS uptake in ~~our~~ study was low despite using a suboptimal cut-off point of 90 days/pills; this is only a third of what WHO recommends. This highlights that the optimal IFAS uptake is significantly lower than what we studied and require immediate action.

Parity was associated with the uptake of IFAS among pregnant women. Primiparous women were less likely to have adequate IFAS uptake compared to multiparous women. Similar findings have been reported in Tanzania, Uganda and other similar contexts (20,24,25). Multiple pregnancies might have increased exposure to health education provided to pregnant women in ANC, leading to a better understanding of the risk of anemia and its complications and more experience and tolerance to iron supplement pills. These might have increased the uptake among those with a previous history of pregnancies.

The number of antenatal visits also influenced the uptake of IFAS among pregnant women in this study. The more frequently a pregnant woman attends the clinic, the more she is exposed to frequent interactions with healthcare workers, and the more she is followed up on IFAS uptake. This increases their access to iron and folic acid supplementation, and more chances to receive

nutritional education. In each visit, a woman would be tested for hemoglobin, and this may be an indicator of the increased need to take IFAS. Similar evidence was reported in a systematic review and meta-analysis study done in Sub-Saharan Africa in 2021 (26). Other studies also supported these findings (20,27). Closer distance to the facility, knowledge of the importance of ANC visits, and good healthcare workers' attitudes also influence the frequency of antenatal visits among pregnant women and hence more opportunities for IFAS (28,29).

Economic status as the key social determinant of health has an effect on the general population's health, as well as among pregnant women. In this study, the wealth index was also an associated factor with IFAS uptake among pregnant women; the wealthier the household is, the more adequate uptake of IFAS is for pregnant women. Higher SES is associated with education level, access to and use of health information and education (30). Moreover, socio-economic disadvantages, including poverty, negatively influence the uptake of many health interventions, including preventive interventions such as IFAS. As reported previously, women with higher incomes may have better access to IFAS supplements due to their stronger financial ability to purchase despite being available for free in most ANC clinics (21,23,30,31).

Women whose households were food secure were more likely to have had adequate IFAS during pregnancy compared to those whose families had severe food insecurity. Women with food insecurity and low economic status may have other priorities, causing less physical access to antenatal care. This may include engagement with economic activities and, hence, lack of time and financial means to visit the antenatal clinics to obtain IFAS tablets. Due to this, they may also lack access to education on the importance of supplementation. Previous evidence shows a disproportionally higher burden of iron deficiency anemia among food-insecure pregnant women (32). Women from households with food insecurity have minimum or no intake of iron and folic acid supplements and may be faced with additional challenges of lacking iron and folic acid-rich foods.

~~Our~~ study reports an update on the key predictors of IFAS supplementation in urban Tanzania. ~~Our~~ findings should be interpreted in the light of the following limitations. Firstly, there is a potential for recall bias as participants were required to remember the days on which they had taken IFAS tablets during pregnancy and the ANC visits they had attended. We mitigated this by comparing such reports with the alternative source (the RCH card) which partly confirmed if the

participants attended ANC to receive IFAS as recommended. Secondly, some of the study participants did not respond to some of the questions hence ~~our~~ study has some missingness which was mitigated by including the non-response rate in sample size calculation. Lastly, there might be social desirability bias since the questions were asked by the researchers (a doctor and a nurse). This might have influenced a positive response by the study participants. This was mitigated by guaranteeing total confidentiality to the study participants and allowing anonymity by using numbers and leaving out any identifiable information. Given that our study was conducted in urban health facilities, the generalizability of our findings may not apply in rural settings.

Conclusion

The uptake of iron and folic acid supplements among pregnant women is still low, only 31.8% of pregnant women had IFAS uptake for 90 days or more during their most recent pregnancy. Addressing this severe challenge in the uptake of life-serving intervention among the identified vulnerable population with a high burden of anemia in Tanzania, efforts should be focused on primiparous women, women with a low number of ANC visits, and households with food insecurity and low wealth quintiles. Since our studies focused only on women who attended at least one ANC visit, we recommend further studies and interventions to address IFAS uptake for pregnant women with poor or no ANC access.

Acknowledgement

The authors would like to thank the medical officers in charge, the administrative office at the Temeke, Mwananyamala, and Amana regional referral hospitals and all study participants.

Authors' contribution

Conceptualisation: CEK, SB, BFS; Data curation: CEK, BJN; Formal analysis: CEK; Methodology: CEK, SB, BJN, BFS; Supervision: SB, BFS; Validation: SB, BJN, BFS; Visualization: CEK; Interpretation of the Data; CEK, BJN, BFS; Writing – original draft: CEK; Writing – review & editing: SB, BJN, BFS. All authors have approved the final version of the manuscript for publication.

Data availability

There are legal restrictions to sharing the datasets; we do not have approval to share data in public

domains. However, data requests from individuals or entities shall be honored upon fulfilling the national regulatory requirements including data transfer agreement (DTA) as advised by the National Research and Ethics Committee (NIMR) of Tanzania. The datasets used and/or analyzed during the current study can be requested by contacting the Muhimbili University of Health and Allied Sciences (MUHAS) Directorate of Research and Publication at drp@muhas.ac.tz

Funding

This study received no funding

Conflicts of interest

The authors declare no competing interests.

REFERENCES

1. Digssie Gebremariam A, Abebaw Tiruneh S, Abebe Abate B, Tadege Engidaw M, Tesfa Asnakew D. Adherence to iron with folic acid supplementation and its associated factors among pregnant women attending antenatal care follow up at Debre Tabor General Hospital, Ethiopia, 2017. PLoS One. 2019;14(1):1–10.
2. Tefera AA, Ibrahim NA, Umer AA. Adherence to iron and folate supplementation and associated factors among women attending antenatal care in public health facilities at Covid-19 pandemic in Ethiopia. PLOS Glob Public Heal [Internet]. 2023;3(1):e0000825. Available from: http://dx.doi.org/10.1371/journal.pgph.0000825
3. Osman MO, Nour TY, Bashir HM, Roble AK, Nur AM, Abdilahi AO. Risk factors for anemia among pregnant women attending the antenatal care unit in selected jigjiga public health facilities, somali region, east ethiopia 2019: Unmatched case–control study. J Multidiscip Healthc. 2020;13:769–77.
4. Natekar P, Deshmukh C, Limaye D, Ramanathan V, Pawar A. A micro review of a nutritional public health challenge: Iron deficiency anemia in India. Clin Epidemiol Glob Heal [Internet]. 2022;14(July 2021):100992. Available from: https://doi.org/10.1016/j.cegh.2022.100992
5. Raman AS, Rao ES, Dei K. Analysis of blood cell parameters in predicting the diagnosis and differentiation of anaemias Abstract : 2018;17(12):58–62.
6. Zhang Q, Lu XM, Zhang M, Yang CY, Lv SY, Li SF, et al. Adverse effects of iron deficiency anemia on pregnancy outcome and offspring development and intervention of three iron supplements. Sci Rep [Internet]. 2021;11(1):1–11. Available from: https://doi.org/10.1038/s41598-020-79971-y
7. Kamau MW, Mirie W, Kimani ST. Maternal knowledge on iron and folic acid supplementation and associated factors among pregnant women in a rural County in Kenya. Int J Africa Nurs Sci [Internet]. 2019;10(September 2018):74–80. Available from: https://doi.org/10.1016/j.ijans.2019.01.005
8. Patti MA, Braun JM, Arbuckle TE, MacFarlane AJ. Associations between folic acid supplement use and folate status biomarkers in the first and third trimesters of pregnancy in the Maternal–Infant Research on Environmental Chemicals (MIREC) Pregnancy Cohort Study. Am J Clin Nutr [Internet]. 2022;116(6):1852–63. Available from: https://doi.org/10.1093/ajcn/nqac235
9. Assefa H, Abebe SM, Sisay M. Magnitude and factors associated with adherence to Iron and folic acid supplementation among pregnant women in Aykel town, Northwest Ethiopia. BMC Pregnancy Childbirth. 2019;19(1):1–8.
10. Sedlander E, Long MW, Bingenheimer JB, Rimal RN. Examining intentions to take iron supplements to inform a behavioral intervention: The Reduction in Anemia through Normative Innovations (RANI) project. PLoS One [Internet]. 2021;16(5 May):1–16. Available from: http://dx.doi.org/10.1371/journal.pone.0249646
11. World Health Organization, Williams a L, van Drongelen W, Lasky RE, Sanderson M, Lai D, et al. Guideline : Daily iron and folic acid supplementation in pregnant women. World Heal Organ. 2012;46:323–9.
12. Zegeye B, Adjei NK, Olorunsaiye CZ, Ahinkorah BO, Ameyaw EK, Seidu AA, et al.

Pregnant women’s decision-making capacity and adherence to iron supplementation in sub-Saharan Africa: a multi-country analysis of 25 countries. BMC Pregnancy Childbirth [Internet]. 2021;21(1):1–10. Available from: https://doi.org/10.1186/s12884-021-04258-7

13. Tsamantioti E, Alfvén T, Hossin MZ, Razaz N. Maternal anaemia and risk of neonatal and infant mortality in low- and middle-income countries: A secondary analysis of 45 national datasets. BMJ Glob Heal. 2025;10(3):1–10.

14. Wang S, Wei J, Wang D, Hua L, Pan D, Fu L, et al. The association between folic acid supplementation, maternal folate during pregnancy and intelligence development in infants: a prospective cohort study. Food Sci Hum Wellness [Internet]. 2021;10(2):197–204. Available from: https://doi.org/10.1016/j.fshw.2021.02.009

15. Ministry of Health Tanzania, Ministry of Health Zanzibar, National Bureau of Statistics, Office of the Chief Government Statistician, The DHS Program (ICF). Tanzania Demographic and Health Survey and Malaria Indicator Survey (TDHS-MIS). Report. 2022;919.

16. Ministry of Health (MoH) [Tanzania Mainland], Ministry of Health (MoH) [Zanzibar], National Bureau of Statistics (NBS), Office of the Chief Government Statistician (OCGS), and ICFMinistry of Health (MoH) [Tanzania Mainland], Ministry of Health (MoH) [Zanz and I. Tanzania Demographic and Health Survey and Malaria Indicator Survey 2022 Key Indicators Report. 2023;1–23.

17. BF Sunguya, Poudel KC, Otsuka K, Yasuoka J, Mlunde LB, Urassa DP, Mkopi NP JM. Undernutrition among HIV-positive children in Dar es Salaam, Tanzania: antiretroviral therapy alone is not enough. BMC Public Heal. 2011;

18. Maria Siega-Riz, A., Herrmann, T.S., Savitz, D.A. and Thorp JM. Frequency of eating during pregnancy and its effect on preterm delivery. Am J Epidemiol. 2001;pp.647-652.

19. Ministry of Health Community Development Gender Elderly and Children; United Republic of Tanzania. Antenatal care guidelines. 2018.

20. Lyoba WB, Mwakatoga JD, Festo C, Mrema J, Elisaria E. Adherence to Iron-Folic Acid Supplementation and Associated Factors among Pregnant Women in Kasulu Communities in North-Western Tanzania. Int J Reprod Med. 2020;2020:1–11.

21. Nisar YB, Dibley MJ, Mir AM. Factors associated with non-use of antenatal iron and folic acid supplements among Pakistani women: A cross sectional household survey. BMC Pregnancy Childbirth. 2014;14(1):1–12.

22. Kamau MW, Mirie W, Kimani S. Compliance with Iron and folic acid supplementation (IFAS) and associated factors among pregnant women: Results from a cross-sectional study in Kiambu County, Kenya. BMC Public Health. 2018;18(1):1–10.

23. Ogundipe O, Hoyo C, Stbye T, Oneko O, Manongi R, Lie RT, et al. Factors associated with prenatal folic acid and iron supplementation among 21,889 pregnant women in Northern Tanzania: A cross-sectional hospital-based study. BMC Public Health. 2012;12(1):1.

24. Moshi FV, Millanzi WC, Mwampagatwa I. Factors Associated With Uptake of Iron Supplement During Pregnancy Among Women of Reproductive Age in Tanzania: an Analysis of Data From the 2015 to 2016 Tanzania Demographic and Health Survey and Malaria Indicators Survey. Front Public Heal. 2021;9(July):1–8.

25. Nimwesiga C, Murezi M, Taremwa IM. Adherence to Iron and Folic Acid Supplementation and Its Associated Factors among Pregnant Women Attending Antenatal Care at Bwindi Community Hospital, Western Uganda. Int J Reprod Med. 2021;2021:1–10.

26. Fite MB, Roba KT, Oljira L, Tura AK, Yadeta TA. Compliance with Iron and Folic Acid Supplementation (IFAS) and associated factors among pregnant women in Sub- Saharan Africa: A systematic review and metaanalysis. PLoS One [Internet]. 2021;16(4 April):1–17. Available from: http://dx.doi.org/10.1371/journal.pone.0249789

27. Wambugu J, Chege P, Muga M. Maternal determinants of non-compliance to folic acid supplementation and iron among pregnant women in Nakuru North Sub-County, Kenya. J Med Heal Sci. 2022;1(1):46–54.

28. Azanaw MM, Gebremariam AD, Dagnaw FT, Yisak H, Atikilt G, Minuye B, et al. Factors associated with numbers of antenatal care visits in rural ethiopia. J Multidiscip Healthc. 2021;14:1403–11.

29. Acup W, Opollo MS, Akullo BN, Musinguzi M, Kigongo E, Opio B, et al. Factors associated with first antenatal care (ANC) attendance within 12 weeks of pregnancy among women in Lira City, Northern Uganda: a facility-based cross-sectional study. BMJ Open. 2023;13(7):1–8.

30. Ba DM, Ssentongo P, Kjerulff KH, Na M, Liu G, Gao X, et al. Adherence to Iron Supplementation in 22 Sub-Saharan African Countries and Associated Factors among Pregnant Women: A Large Population-Based Study. Curr Dev Nutr. 2019;3(12):1–8.

31. Bancha B, Lajore BA, Petros L, Hassen H, Jemal A. Time to non-adherence to iron and folic acid supplementation and associated factors among pregnant women in Hosanna town, South Ethiopia: Cox-proportional hazard model. PLoS One [Internet]. 2022;17(9 September):1–13. Available from: http://dx.doi.org/10.1371/journal.pone.0275086

32. Park CY, Eicher-Miller HA. Iron Deficiency Is Associated with Food Insecurity inPregnant Females in the United States: National Health and Nutrition Examination Survey 1999-2010. J Acad Nutr Diet. 2014;114(12):1967–73.

Iron and folic acid supplements uptake among pregnant women in Dar es Salaam, Tanzania

Careen E. Koka[1], Saida Bakari[1], Belinda J. Njiro[1*], Bruno F. Sunguya[1]

[1]School of Public Health and Social Sciences, Muhimbili University of Health and Allied Sciences, 9 United Nations Road, Dar es Salaam, Tanzania

[*]Corresponding Author: Belinda J. Njiro
Email: belindaj.njiro@gmail.com

Department of Epidemiology and Biostatistics, School of Public Health and Social Sciences, Muhimbili University of Health and Allied Sciences.
Address: 9 United Nations Road, Upanga West, P.O Box 65001, Dar es Salaam, Tanzania
Tel: +255 755 226 469

Authors emails:
CEK: careenkoka@icloud.com
SB: saidahmohamed2014@gmail.com
BJN: belindaj.njiro@gmail.com
BFS: sunguya@gmail.com; bruno.sunguya@muhas.ac.tz

Abstract

Background: Anemia in pregnancy is a public health concern globally with the biggest brunt felt in low and middle-income countries (LMICs). Tanzania is no exception. Iron and folic acid (~~IFA~~IFAS) intake is a proven intervention recommended to address the deficiencies behind anemia in pregnancy. Despite interventions in Tanzania, ~~IFA~~IFAS uptake has remained low owing to various reasons not well documented.

Materials and Methods: A quantitative cross-sectional study was conducted to assess levels and determinants of ~~IFA~~IFAS uptake during pregnancy in Dar es Salaam, Tanzania. Data was collected among 428 post-delivery women in postnatal wards of Temeke, Mwananyamala, and Amana regional referral hospitals. Descriptive analyses were conducted to determine levels and characteristics of ~~IFA~~IFAS uptake, whereas bivariate and multivariate analyses were conducted to examine determinants of uptake using SPSS version 23.

Result: About one in three pregnant women (31.8%) who attended antenatal care (ANC) at referral hospitals had an appropriate IFAS uptake. Adequate IFAS uptake was 74% lower among primiparous women compared to multiparous women (aOR=0.26; 95% CI: 0.10 – 0.67). Women who reported five or more ANC visits (aOR=11.7; 95%CI: 1.30 – 63.66) and women from high wealth index families index (aOR=11.68; 95% CI: 2.91 – 89.57) were 12 times more likely to have adequate IFAS uptake than those with fewer visits respectively. Living in a food-secure household was also associated with higher IFAS uptake by nine times compared to women whose households had severe food insecurity (aOR=9.21; 95% CI: 1.82 – 10.33).

Conclusion: Only 31.8% of pregnant women attending regional referral hospitals in Dar es Salaam had appropriate IFAS uptake to prevent anaemia during pregnancy. Such unprecedented low uptake needs to be addressed through targeted interventions among pregnant women with higher parity, fewer ANC attendances, challenging family support, and coming from households with economic hardship and food insecurity.

Keywords: *Iron and folic acid, micronutrient supplementation, pregnant women, anemia, anemia in pregnancy, Tanzania*

Introduction

Anemia in pregnancy is diagnosed as hemoglobin concentration of less than 11.0 g/dl in the first trimester, 10.5g/dl in the second trimester and 11.0g/dl in the third trimester (1). ~~It is usually caused by iron deficiency~~Iron deficiency is the most common cause of Anemia in women of reproductive ages and in pregnancy (1). Iron is a crucial component of hemoglobin, a protein found in red blood cells (RBCs) that is responsible for carrying oxygen throughout the body.~~The red blood cell (RBC) protein,hemoglobin, which carries oxygen from the lungs to the tissues, is significantly dependent on iron.~~ Human bodies need folic acid for the synthesis, repair, methylation, and cell division of DNA. Folic acid is also needed to develop healthy RBCs and to prevent anemia, especially during pregnancy and infancy (2).

Nutritional anemia is the most common type of anemia in pregnancy and is a significant public health challenge worldwide, particularly in low- and middle-income countries (LMICs)(3). Anemia affects about1.62 billion people globally, accounting for 24.8% of the world's population (4,5). Pregnant women are the most vulnerable populations in nutritional anemia (6); 56.4 million pregnant women are affected by anemia, and of them 17.2 million are from Africa (1). Iron deficiency anemia, accounts for more than half of all occurrences of anemia in pregnancy, which is one of the major contributing factors to the worldwide burden of disease (7).

Pregnancy-related physiological changes, fetal growth, and development raise the need for iron and folic acid (8). Due to the lower bioavailability of nutrients in pregnant women, the increased demand for essential nutrients cannot be satisfied by diet alone (9). If nutrition is not supplemented with iron and folic acid tablets throughout pregnancy, there is a considerable risk that iron deficiency and folic acid deficiency may manifest (9). The World Health Organization (WHO) recommends iron and folic acid supplementation (IFAS) as one of the interventions~~isone of theinterventionsby the WHO~~ for the control of anemia in pregnancy (1,7,10).

The ~~World Health Organization (~~WHO~~)~~ recommends that pregnant women take daily doses of 30 to 60 mg of iron and 0.4 mg of folic acid to prevent maternal anemia (11). ~~,~~Iron and folic acid deficiency and anemia in pregnancy have been linked with maternal ~~mobidity~~morbidity including fatigue, breathing difficulties, higher risk of postpartum depression, blood loss during labor, and ~~,~~infections including puerperal sepsis. Negative consequences for the infants include~~,~~ low birth

weight, neural tube defects such as spina bifida and anencephaly, intrauterine growth restriction, impaired fetal growth, ~~and~~ preterm birth, and perinatal mortality (11–13)~~(11,12)~~. The risk of all types of maternal anemia at term is reduced by 70% and iron deficiency anemia at term by 57%, if daily iron and folic acid supplements (IFAS) are taken throughout pregnancy (1). The risk of infant mortality can also be decreased by 34% if a pregnant woman takes IFAS for 90 days during pregnancy (1). Moreover, the levels of maternal folic acid may also influence infants' intellectual development (14).

To attain a 50% reduction in anemia among women of reproductive age by 2025, the WHO recommends the provision of IFAS in pregnancy (2). Reduced maternal and child morbidity and mortality are already notable with the use of IFAS (7). However, the uptake has remained low in many regions and countries, including Tanzania. Evidence on the associated factors varies and sometimes lacking in some contexts including Tanzania. This study aimed to fill this evidence gap by documenting Iron and folic acid supplement uptake and associated factors among pregnant women in Dar es Salaam, Tanzania

Methodology

Study design and setting

This hospital-based cross-sectional quantitative study was conducted to determine the factors associated with iron and folic acid supplement uptake among pregnant women in Dar es Salaam, Tanzania. The study was conducted from 1st to 31st of July 2024 at three referral hospitals in Dar es Salaam, namely, Temeke, Mwananyamala and Amana regional referral hospitals located in Temeke, Ilala, and Kinondoni districts respectively.

Study population

Data was collected from post-natal women attending the three health facilities at their respective post-natal wards. Both post-spontaneous vaginal delivery and post-cesarean section women that gave birth were eligible to participate and were involved in the study. All post-natal women aged 15 to 49 years visiting the post-natal wards at Temeke, Amana, and Mwananyamala regional referral hospitals were eligible to participate. We included only women with a prior history of antenatal care visits to any health facility during their most recent pregnancy and who had their antenatal care (ANC) cards for quality checks. The study excluded women who were in severe pain, had health emergencies and those who did not consent to participate in the study.

Sampling and sample size

The hospitals were purposively selected owing to the volume of the clients and therefore sample population. Participants were randomly selected for the study using a simple random sampling technique. The sample size was estimated by using Fisher's formula $N=Z^2 P (1-P) / E^2$, where N is the estimated minimum sample size; Z is the confidence level at 95% (standard value is 1.96); P is the proportion (prevalence of iron and folic acid adherence (50%); and E is the precision at 95% CI = 0.05. The minimum sample that was required for this study was 385 post-natal women. A 10% non-response rate was used to give a total sample size of 428 post-natal women. Participants were selected through a simple random sampling technique using a lottery technique whereby participants were assigned numbers each. Numbers were written on small pieces of paper, of the same size, color and shape. The papers were folded and then put in a box and then mixed. A blindfold selection was made. The process of selecting continued until the required sample size

per respective hospital was reached. About 18 – 24 participants were selected in a day.

Data collection and measurements

Data was collected from respondents via face-to-face interviews by the researcher and two trained research assistants using a semi-structured questionnaire adopted from the 2022 Tanzania Demographic Health Survey (TDHS)~~2022~~ and previous studies (15,16)~~.~~ All the questions were prepared in English and translated in Swahili (local language). The interviews lasted for approximately 30 minutes for each respondent; data collection was conducted at the respondents' respective postnatal wards. We collected data on maternal age, education level, employment status, and wealth index. Weighted wealth index was used as a proxy for household economic level. It considered the ownership of home resources, dwelling features, fuel for cooking and lighting, toilet type, water supply, and feeding habits was used to determine economic status. The items were weighted and added together to produce the wealth index. The weighted wealth index score was split similarly into three (terciles) representing high, medium, and low economic levels. Maternal factors assessed included parity, timing of the first ANC, and number of ANC visits. Health facility factors included distance to the health facility and HCP's attitude.

The feeding frequency tool was adopted from previous Tanzanian research projects conducted in Tanzania (17).The ~~The World Health Organization~~ WHO suggests a feeding schedule of three meals and at the minimum two snacks in 24 hours for pregnant women ~~(WHO)~~ (18). The dietary Diversity Scale (DDS) was used to measure dietary diversity adopted from previous studies conducted in Tanzania. We extracted a roster of twelve foods from the 2022 Tanzania Demographic Health Survey (TDHS) (15). Participants were asked to mention the food items they had on the earlier day, and we determined the final score for dietary diversification. Dietary diversity below 4 was considered as low. Household food insecurity was assessed using a nine-item Household Food Insecurity Access Score (HFIAS) tool. On this scale, the options are: 0 ="no," 1 = "infrequently," 2 = "spoken" and 3 = "frequently"." The total score ranges from 0 to 27. The food insecurity magnitude can be classified into four categories: highly insecure (18 – 27), severely insecure (11 – 17), mildly insecure (2 – 10), and secure (0 – 1).

The primary outcome for this study was the ~~IFA~~IFAS uptake of 90 pills or more. ~~IFA~~IFAS uptake was self-reported by the study participants as the average number of days that participants took

iron and folic acid supplements during their most recent pregnancy (15). This information was double-checked on the Reproductive and Child Health (RCH) card used to record maternal and child assessments and progress during pregnancy and postpartum periods by the healthcare workers. In Tanzania, all pregnant women receive a single pill containing 200mg of ferrous sulphate and 0.4 mg of folic acid daily (19); this is equivalent to 65~~30-60~~ mg of elementary iron and 400 μg of folic acid daily starting at the first antenatal visit as WHO recommends (11). WHO recommends a daily intake of iron and folic acid throughout the pregnancy (11). We~~We, therefore,~~ defined adequate ~~IFA~~IFAS uptake as the intake of folic acid and iron tablets for a minimum of 90 days per pregnancy adopted from previous studies in similar settings from Tanzania and Pakistan, and using TDHS methodologies (1,20,21). We analyzed IFAS uptake as a categorical variable categorized as normal uptake (90 or more~~-180~~ pills per pregnancy) and low uptake (less than 90 pills per pregnancy). The adequate IFAS uptake in our study is equivalent to at least a third of the WHO recommended daily IFAS intake throughout pregnancy (20,21). ~~Adequate IFAIFAS uptake is defined as four IFAIFAS tablets every week for the duration of the pregnancy~~.

Data analysis

The collected data was analyzed to identify the factors associated with low IFAS uptake using both descriptive and logistic regression analyses. We employed descriptive analysis to summarize the study population characteristics. We analyzed IFAS uptake as a categorical variable with levels, namely adequate uptake (90 pills or more per pregnancy) and low uptake (less than 90 pills per pregnancy). The magnitude of IFAS uptake was calculated as the percentage of IFAS uptake of 90 pills or moreover the total study population. The Pearson Chi-square test was used to examine the differences in the proportions of IFAS uptake by participants' characteristics. Bivariate and Multivariate logistic regression analyses were computed, and crude and adjusted odds ratios with corresponding 95% confidence intervals were recorded. Variables were included in the final model if having a p-value of <0.2 in the bivariate analysis. All analyses were conducted using IBM SPSS version 23.0. A p-value of <0.05 was considered statistically significant.

Ethical considerations

This study received ethical clearance from the Institutional Review Board of the Muhimbili University of Health and Allied Science (MUHAS IRB); ethical approval number MUHAS-REC-

06-2024-2348. We sought the necessary permissions from the district administrative office and the respective hospitals at Temeke, Mwananyamala, and Amana regional referral hospitals. All participants provided written consent to participate before inclusion in the study. For participants aged less than 18 years, we obtained written assent; informed verbal consent was also obtained from their respective guardians. The information obtained from the participants was used for research purposes alone and are available to authorized personnel only.

Patient and Public Involvement

Patient or the public were not directly involved in the design and conduct of this research. However, as part of our dissemination plan, the final report from our analysis will be shared with the included health facilities to inform them of the status of ~~IFA~~IFAS uptake and the relevant recommendations.

Results

~~Participants~~Participant's characteristics

A total of 428 participants (post-delivery women) were recruited for the study with full informed consent. **Table 1** shows the participants' characteristics stratified by the adequacy of ~~IFA~~IFAS uptake. Among all participants, only 31.8% (n=136) had adequate ~~IFA~~IFAS uptake during pregnancy. The highest proportion of adequate ~~IFA~~IFAS uptake was among participants with university-level education (53.8%, n=28), multiparous women (42.0%, n=58), women who booked their ANC visit in the first trimester (53.5%, n=106), and those that had five or more ANC visits. The proportion of adequate ~~IFA~~IFAS uptake was lower among women in the low wealth index and those who reported experiencing poor healthcare provider attitudes.

Table 1: Uptake of IFAS by participants' characteristics (N=428)

Variables	Total n (%)~~N=428~~	Adequate uptake, n (%)	Inadequate uptake, n (%)	*p-value*
**Age groups**				0.688
16 – 25	188 (43.9)	56 ~~(29.8)~~(41.2)	132 ~~(70.2)~~(45.2)	
26 – 35	194 (45.3)	62 ~~(32)~~ (45.5)	132 ~~(68)~~ (45.2)	
36 – 45	46 (10.7)	18 ~~(39.1)~~ (13.2)	28 ~~(60.9)~~(9.6)	
**Highest education level**				
Primary level	142 (33.2)	52 ~~(36.6)~~(34.7)	90 ~~(63.4)~~(33.1)	0.027
Secondary level	228 (53.3)	70 ~~(30.7)~~(46.7)	158 ~~(69.3)~~(58.1)	
University	52 (12.1)	28 ~~(53.8)~~(18.7)	24 ~~(46.2)~~(8.8)	
**Parity**				
Primiparity	164 (38.3)	38 ~~(23.2)~~(27.9)	126 ~~(76.8)~~(43.2)	0.046
Secundiparity	126 (29.4)	40 ~~(31.7)~~(29.4)	86~~(68.3)~~(29.5)	
Multiparity	138 (32.2)	58 ~~(42.0)~~(42.6)	80~~(58.0)~~(27.4)	
**Time of first booking**				
1st trimester	198 (46.3)	106 ~~(53.5)~~(70.7)	92 ~~(46.5)~~(33.8)	0.003
2nd& 3^{rd}trimester	200 (46.7)	44 ~~(22)~~(28.2)	156(57.4)	
Don't know	30 (7.0)	6 ~~(20)~~(8.8)	24 ~~(80)~~(8.8)	
**Number of ANC visits**				
1 – 4	168 (39.3)	24 ~~(14.3)~~(17.6)	144 ~~(85.7)~~(49.3)	<0.001
5 and above	212 (49.5)	104 ~~(49.1)~~(76.5)	108 ~~(50.9)~~(37.0)	
Don't know	48 (11.2)	8 ~~(16.7)~~(5.9)	40 ~~(83.3)~~(13.7)	
**Wealth index**				

Formatted: Font color: Text 1
Formatted: Font: (Default) Times New Roman, 12 pt, Not Italic, Font color: Text 1
Formatted: Font: 11 pt
Formatted Table
Formatted: Font: 11 pt
Formatted: Font: 11 pt
Formatted: Font: 11 pt
Formatted: Font: 11 pt
Formatted: Font: 11 pt
Formatted: Font: 11 pt
Formatted: Font: 11 pt
Formatted: Font: 11 pt
Formatted: Font: 11 pt
Formatted: Font: 11 pt
Formatted: Font: 11 pt
Formatted: Font: 11 pt
Formatted: Font: 11 pt
Formatted: Font: 11 pt
Formatted: Font: 11 pt
Formatted: Font: 11 pt
Formatted: Font: 11 pt
Formatted: Font: 11 pt
Formatted: Font: 11 pt
Formatted: Font: 11 pt
Formatted: Font: 11 pt
Formatted: Font: 11 pt
Formatted: Font: 11 pt
Formatted: Font: 11 pt

High	166 (38.8)	74 ~~(44.6)~~(54.4)	92 ~~(55.4)~~(31.5)	0.001
Middle	173 (40.4)	55 ~~(31.8)~~(40.4)	118 ~~(68.2)~~(40.4)	
Low	89 (20.8)	7 ~~(7.9)~~(5.1)	82 ~~(92.1)~~(28.1)	
**Distance to health facility affecting treatment**				
Yes	158 (36.9)	36 ~~(22.7)~~(26.5)	122 ~~(77.2)~~(41.8)	0.031
No	270 (63.1)	100 ~~(37)~~(73.5)	170 ~~(63)~~(58.2)	
**Healthcare worker's attitude**				
Good	292 (68.2)	106 ~~(36.3)~~(77.9)	186 ~~(63.7)~~(63.7)	0.037
Fair/bad	136 (31.8)	30 ~~(22.1)~~(22.1)	106 ~~(77.9)~~(36.3)	
**Household food security**				0.011
Food secure	226 (52.8)	95 ~~(42)~~(69.9)	131 ~~(58)~~(44.9)	
Mildly insecure	30 (7.0)	11 ~~(36.7)~~(8.1)	19 ~~(63.3)~~(6.5)	
Moderately insecure	77 (18.0)	15 ~~(19.5)~~(11.0)	62 ~~(80.5)~~(21.2)	
Severely insecure	95 (22.2)	15 ~~(15.8)~~(11.0)	80 ~~(84.2)~~(27.4)	
**Dietary diversity score**				0.159
Below 4	208 (48.6)	55 ~~(26.4)~~(40.4)	153 ~~(73.6)~~(52.4)	
4 and above	220 (51.4)	81 ~~(36.8)~~(59.6)	139 ~~(63.2)~~(47.6)	
**Feeding frequency**				0.002
Low	143 (33.4)	22 ~~(15.4)~~(16.2)	121 ~~(84.6)~~(41.4)	
Good	285 (66.6)	114 ~~(40)~~(83.8)	171 ~~(60)~~(58.6)	

Factors associated with adequate iron and folic acid uptake

Table 2 shows the bivariate and multiple logistic regression analysis findings. The odds of adequate IFAS uptake were 74% lower among primiparous women as compared to multiparous women (aOR=0.26; 95% CI: 0.10 – 0.67). Women who reported five or more antenatal visits were 12 times more likely to have adequate IFAS uptake than those who attended 1–4 ANC visits (aOR=11.7; 95% CI: 1.62 – 84.34). Women from high-wealth index families were12 times more likely to report adequate IFAS uptake than women whose families had low wealth index (aOR=11.68; 95% CI: 2.91 – 89.57). Living in a food-secure household was also associated with higher IFAS uptake by nine times compared to women whose households had severe food insecurity (aOR=9.21; 95% CI: 1.82 – 10.33).

Formatted: Font: 11 pt
Formatted: Font: 11 pt
Formatted: Line spacing: single
Formatted: Font: 11 pt
Formatted: Line spacing: single
Formatted: Font: 11 pt
Formatted: Line spacing: single
Formatted: Font: 11 pt
Formatted: Font: 11 pt
Formatted: Line spacing: single
Formatted: Font: 11 pt
Formatted: Line spacing: single
Formatted: Font: 11 pt
Formatted: Font: 11 pt
Formatted: Line spacing: single
Formatted: Font: 11 pt
Formatted: Font: 11 pt
Formatted: Line spacing: single
Formatted: Font: 11 pt
Formatted: Line spacing: single
Formatted: Font: 11 pt
Formatted: Line spacing: single
Formatted: Font: 11 pt
Formatted: Font: 11 pt
Formatted: Line spacing: single
Formatted: Font: 11 pt
Formatted: Line spacing: single
Formatted: Font: 11 pt
Formatted: Font: 11 pt
Formatted: Line spacing: single
Formatted: Font: 11 pt

Table 2: Bivariate and multivariate analysis for factors associated with ~~IFA~~IFAS uptake

Variable	Bivariate analysis		Multivariate analysis	
	**cOR (95%CI)**	***p value***	**aOR (95%CI)**	***p value***
**Highest education level**				
Primary	0.29 (0.11 – 0.74)	0.010	0.54 (0.16 – 1.91)	0.341
Secondary	0.38 (0.16 – 0.90)	0.029	0.65 (0.20 – 2.11)	0.474
University	Reference		Reference	
**Parity**				
Primiparity	0.42 (0.21 – 0.84)	0.014	0.26 (0.10 – 0.67)	0.005
Secundiparity	0.64 (0.31 – 1.31)	0.223	0.58 (0.23 – 1.47)	0.246
Multiparity	Reference		Reference	
**Time of first booking**				
1st trimester	3.07(0.82 – 11.57)	0.097	0.82 (0.09 – 7.19~~s~~)	0.857
2nd& 3rdtrimester	1.13 (0.29 – 4.26)	0.861	0.54 (0.06 – 4.95)	0.585
Don't know	Reference		Reference	
**Number of ANC visits**				
1 – 4	0.83 (0.24 – 2.87)	0.772	2.23 (0.29 – 16.86)	0.439
5 and above	4.82 (1.54 – 15.04)	0.007	11.70 (1.30 – 63.66)	0.015
Don't know	Reference		Reference	
**Wealth index**				
High	16.25 (3.0 – 88.9)	0.001	11.68 (2.91 – 89.57)	0.059
Middle	6.96 (1.3 – 36.5)	0.022	6.59 (0.69–62.96)	0.102
Low	Reference		Reference	
**Distance to health facility**				
Yes	1.99 (1.06 – 3.75)	0.032	1.92 (0.82 – 4.51)	0.135
No	Reference		Reference	
**Health care workers attitude**				
Good	0.50 (0.26 – 0.97)	0.039	0.62 (0.25 – 1.56)	0.313
Fair/bad	Reference		Reference	
**Household food security**				
Food secure	6.50 (1.73 – 24.39)	0.006	9.21 (1.82 – 10.33)	0.047
Mild food insecurity	4.50 (0.54 – 37.38)	0.164	8.96 (0.16 – 13.13)	0.234
Moderate food insecurity	1.33 (0.26 – 6.83)	0.730	4.15 (0.47 – 6.24)	0.820
Severe food insecurity	Reference		Reference	
**Dietary diversity score**				
<4 (low)	1.96 (0.77 – 4.99)	0.161	0.73 (0.21 – 2.51)	0.617
4 and above (good)	Reference		Reference	
**Food frequency**				
Low	5.47 (1.83 –16.39)	0.002	1.75 (0.31 – 9.82)	0.526

Good	Reference	Reference

aOR: adjusted odds ratio, cOR: crude odds ratio, ANC: antenatal care

Discussion

Less than one in three women had adequate of iron and folic acid supplementation during pregnancy in Dar es Salaam, Tanzania. Inadequate IFAS uptake in the context of the high burden of anemia among pregnant women calls for immediate and sustained efforts to address the burden of anemia in pregnancy. In Tanzania, similar results were ~~also recently~~ documented in the 2022 TDHS~~Mbeya region~~, ~~whereby the uptake was~~where the IFAS uptake for 90 days and more was reported at~~of~~ 40.8% (16). ~~. of 37.8%~~ ~~(22)~~ ~~and~~ Our IFAS uptake findings are also not very different from evidence from a neighboring country, Kenya (22). The current findings show an improvement compared to a previous study conducted in Moshi Tanzania where ~~the~~ 16.1% of women reported taking folic acid and iron supplements~~uptake documented~~ in 2009 ~~from Moshi, Tanzania~~(23). This study, however, reported on any use of IFA supplements and did not report on the adequacy of intake. The slight improvement reported could be attributed to improved ANC attendance, access to nutritional ~~counselling~~counseling at the antenatal clinics, increased number of health facilities and trained health workers, and campaigns on facility-based child deliveries by the government and implementing partners. It is worth noting that the adequacy of IFAS uptake in our study was low despite using a suboptimal cut-off point of 90 days/pills; this is only a third of what WHO recommends. This highlights that the optimal IFAS uptake is significantly lower than what we studied and require immediate action.

Parity was associated with the uptake of IFAS among pregnant women. Primiparous women were less likely to have adequate ~~IFA~~IFAS uptake compared to multiparous women. Similar findings have been reported in Tanzania, Uganda and other similar contexts (20,24,25). Multiple pregnancies might have increased exposure to health education provided to pregnant women in ANC, leading to a better understanding of the risk of anemia and its complications and more experience and tolerance to iron supplement pills. These might have increased the uptake among those with a previous history of pregnancies.

The number of antenatal visits also influenced the uptake of IFAS among pregnant women in this study. The more frequently a pregnant woman attends the clinic, the more she is exposed to

frequent interactions with healthcare workers, and the more she is followed up on IFAS uptake. This increases their access to iron and folic acid supplementation, and more chances to receive nutritional education. In each visit, a woman would be tested for hemoglobin, and this may be an indicator of the increased need to take IFAS. Similar evidence was reported in a systematic review and meta-analysis study done in Sub-Saharan Africa in 2021 (26). Other studies also supported these findings (20,27). Closer distance to the facility, knowledge of the importance of ANC visits, and good healthcare workers' attitudes also influence the frequency of antenatal visits among pregnant women and hence more opportunities for IFAS (28,29).

Economic status as the key social determinant of health has an effect on the general population's health, as well as among pregnant women. In this study, the wealth index was also an associated factor with IFAS uptake among pregnant women; the wealthier the household is, the more adequate uptake of IFAS is for pregnant women. Higher SES is associated with education level, access to and use of health information and education (30). Moreover, socio-economic disadvantages, including poverty, negatively influence the uptake of many health interventions, including preventive interventions such as IFAS. As reported previously, women with higher incomes may have better access to ~~IFA~~IFAS supplements due to their stronger financial ability to purchase despite being available for free in most ANC clinics (21,23,30,31).

Women whose households were food secure were more likely to have had adequate IFAS during pregnancy compared to those whose families had severe food insecurity. Women with food insecurity and low economic status may have other priorities, causing less physical access to antenatal care. This may include engagement with economic activities and, hence, lack of time and financial means to visit the antenatal clinics to obtain ~~IFA~~IFAS tablets. Due to this, they may also lack access to education on the importance of supplementation. Previous evidence shows a disproportionally higher burden of iron deficiency anemia among food-insecure pregnant women (32). Women from households with food insecurity have minimum or no intake of iron and folic acid supplements and may be faced with additional challenges of lacking iron and folic acid-rich foods.

Our study reports an update on the key predictors of ~~IFA~~IFAS supplementation in urban Tanzania. Our findings should be interpreted in the light of the following limitations. Firstly, there is a potential for recall bias as participants were required to remember the days on which they had

Formatted

taken IFAS tablets during pregnancy and the ANC visits they had attended. ~~First,there is a risk of recall bias since participants had to recall the days they had taken IFAS tablets they had takenduring pregnancy and ANC visits they had attended.~~We mitigated this by comparing such reports with the alternative source (the RCH card) which partly confirmed if the participants attended ANC to receive IFAS as recommended. Secondly, some of the study participants did not respond to some of the questions hence our study has some missingness which was mitigated by including the non-response rate in sample size calculation. Lastly, there might be social desirability bias since the questions were asked by the researchers (a doctor and a nurse). This might have influenced a positive response by the study participants. This was mitigated by guaranteeing total confidentiality to the study participants and allowing anonymity by using numbers and leaving out any identifiable information. Given that our study was conducted in urban health facilities, the generalizability of our findings may not apply in rural settings.

Formatted: Font: 12 pt

Formatted

Conclusion

The uptake of iron and folic acid supplements among pregnant women is still low, only 31.8% of pregnant women had ~~adequate~~ IFAS uptake for 90 days or more ~~in Dar es Salaam, Tanzania~~during their most recent pregnancy. Addressing this severe challenge in the uptake of life-serving intervention among the identified vulnerable population with a high burden of anemia in Tanzania, efforts should be focused on primiparous women, women with a low number of ANC visits, and households with food insecurity and low wealth quintiles. Since our studies focused only on women who attended at least one ANC visit, we recommend further studies and interventions to address IFAS uptake for pregnant women with poor or no ANC access.

Acknowledgement

The authors would like to thank the medical officers in charge, the administrative office at the Temeke, Mwananyamala, and Amana regional referral hospitals and all study participants.

Authors' contribution

Conceptualisation: CEK, SB, BFS; Data curation: CEK, BJN; Formal analysis: CEK; Methodology: CEK, SB, BJN, BFS; Supervision: SB, BFS; Validation: SB, BJN, BFS; Visualization: CEK; Interpretation of the Data; CEK, BJN, BFS; Writing – original draft: CEK;

Writing – review & editing: SB, BJN, BFS. All authors have approved the final version of the manuscript for publication.

Data availability

There are legal restrictions to sharing the datasets; we do not have approval to share data in public domains. However, data requests from individuals or entities shall be honored upon fulfilling the national regulatory requirements including data transfer agreement (DTA) as advised by the National Research and Ethics Committee (NIMR) of Tanzania. The datasets used and/or analyzed during the current study can be requested by contacting the Muhimbili University of Health and Allied Sciences (MUHAS) Directorate of Research and Publication at drp@muhas.ac.tz

~~Data described in the manuscript, code book, and analytic codes will be made available upon request.~~

Funding

This study received no funding

Conflicts of interest

The authors declare no competing interests.

REFERENCES

1. Digssie Gebremariam A, Abebaw Tiruneh S, Abebe Abate B, Tadege Engidaw M, Tesfa Asnakew D. Adherence to iron with folic acid supplementation and its associated factors among pregnant women attending antenatal care follow up at Debre Tabor General Hospital, Ethiopia, 2017. PLoS One. 2019;14(1):1–10.
2. Tefera AA, Ibrahim NA, Umer AA. Adherence to iron and folate supplementation and associated factors among women attending antenatal care in public health facilities at Covid-19 pandemic in Ethiopia. PLOS Glob Public Heal [Internet]. 2023;3(1):e0000825. Available from: http://dx.doi.org/10.1371/journal.pgph.0000825
3. Osman MO, Nour TY, Bashir HM, Roble AK, Nur AM, Abdilahi AO. Risk factors for anemia among pregnant women attending the antenatal care unit in selected jigjiga public health facilities, somali region, east ethiopia 2019: Unmatched case–control study. J Multidiscip Healthc. 2020;13:769–77.
4. Natekar P, Deshmukh C, Limaye D, Ramanathan V, Pawar A. A micro review of a nutritional public health challenge: Iron deficiency anemia in India. Clin Epidemiol Glob Heal [Internet]. 2022;14(July 2021):100992. Available from: https://doi.org/10.1016/j.cegh.2022.100992
5. Raman AS, Rao ES, Dei K. Analysis of blood cell parameters in predicting the diagnosis and differentiation of anaemias Abstract : 2018;17(12):58–62.
6. Zhang Q, Lu XM, Zhang M, Yang CY, Lv SY, Li SF, et al. Adverse effects of iron deficiency anemia on pregnancy outcome and offspring development and intervention of three iron supplements. Sci Rep [Internet]. 2021;11(1):1–11. Available from: https://doi.org/10.1038/s41598-020-79971-y
7. Kamau MW, Mirie W, Kimani ST. Maternal knowledge on iron and folic acid supplementation and associated factors among pregnant women in a rural County in Kenya. Int J Africa Nurs Sci [Internet]. 2019;10(September 2018):74–80. Available from: https://doi.org/10.1016/j.ijans.2019.01.005
8. Patti MA, Braun JM, Arbuckle TE, MacFarlane AJ. Associations between folic acid supplement use and folate status biomarkers in the first and third trimesters of pregnancy in the Maternal–Infant Research on Environmental Chemicals (MIREC) Pregnancy Cohort Study. Am J Clin Nutr [Internet]. 2022;116(6):1852–63. Available from: https://doi.org/10.1093/ajcn/nqac235
9. Assefa H, Abebe SM, Sisay M. Magnitude and factors associated with adherence to Iron and folic acid supplementation among pregnant women in Aykel town, Northwest Ethiopia. BMC Pregnancy Childbirth. 2019;19(1):1–8.
10. Sedlander E, Long MW, Bingenheimer JB, Rimal RN. Examining intentions to take iron supplements to inform a behavioral intervention: The Reduction in Anemia through Normative Innovations (RANI) project. PLoS One [Internet]. 2021;16(5 May):1–16. Available from: http://dx.doi.org/10.1371/journal.pone.0249646
11. World Health Organization, Williams a L, van Drongelen W, Lasky RE, Sanderson M, Lai D, et al. Guideline : Daily iron and folic acid supplementation in pregnant women. World Heal Organ. 2012;46:323–9.
12. Zegeye B, Adjei NK, Olorunsaiye CZ, Ahinkorah BO, Ameyaw EK, Seidu AA, et al.

Pregnant women's decision-making capacity and adherence to iron supplementation in sub-Saharan Africa: a multi-country analysis of 25 countries. BMC Pregnancy Childbirth [Internet]. 2021;21(1):1–10. Available from: https://doi.org/10.1186/s12884-021-04258-7

13. Tsamantioti E, Alfvén T, Hossin MZ, Razaz N. Maternal anaemia and risk of neonatal and infant mortality in low- and middle-income countries: A secondary analysis of 45 national datasets. BMJ Glob Heal. 2025;10(3):1–10.

14. Wang S, Wei J, Wang D, Hua L, Pan D, Fu L, et al. The association between folic acid supplementation, maternal folate during pregnancy and intelligence development in infants: a prospective cohort study. Food Sci Hum Wellness [Internet]. 2021;10(2):197–204. Available from: https://doi.org/10.1016/j.fshw.2021.02.009

15. Ministry of Health Tanzania, Ministry of Health Zanzibar, National Bureau of Statistics, Office of the Chief Government Statistician, The DHS Program (ICF). Tanzania Demographic and Health Survey and Malaria Indicator Survey (TDHS-MIS). Report. 2022;919.

16. Ministry of Health (MoH) [Tanzania Mainland], Ministry of Health (MoH) [Zanzibar], National Bureau of Statistics (NBS), Office of the Chief Government Statistician (OCGS), and ICFMinistry of Health (MoH) [Tanzania Mainland], Ministry of Health (MoH) [Zanz and I. Tanzania Demographic and Health Survey and Malaria Indicator Survey 2022 Key Indicators Report. 2023;1–23.

17. BF Sunguya, Poudel KC, Otsuka K, Yasuoka J, Mlunde LB, Urassa DP, Mkopi NP JM. Undernutrition among HIV-positive children in Dar es Salaam, Tanzania: antiretroviral therapy alone is not enough. BMC Public Heal. 2011;

18. Maria Siega-Riz, A., Herrmann, T.S., Savitz, D.A. and Thorp JM. Frequency of eating during pregnancy and its effect on preterm delivery. Am J Epidemiol. 2001;pp.647-652.

19. Ministry of Health Community Development Gender Elderly and Children; United Republic of Tanzania. Antenatal care guidelines. 2018.

20. Lyoba WB, Mwakatoga JD, Festo C, Mrema J, Elisaria E. Adherence to Iron-Folic Acid Supplementation and Associated Factors among Pregnant Women in Kasulu Communities in North-Western Tanzania. Int J Reprod Med. 2020;2020:1–11.

21. Nisar YB, Dibley MJ, Mir AM. Factors associated with non-use of antenatal iron and folic acid supplements among Pakistani women: A cross sectional household survey. BMC Pregnancy Childbirth. 2014;14(1):1–12.

22. Kamau MW, Mirie W, Kimani S. Compliance with Iron and folic acid supplementation (IFAS) and associated factors among pregnant women: Results from a cross-sectional study in Kiambu County, Kenya. BMC Public Health. 2018;18(1):1–10.

23. Ogundipe O, Hoyo C, Stbye T, Oneko O, Manongi R, Lie RT, et al. Factors associated with prenatal folic acid and iron supplementation among 21,889 pregnant women in Northern Tanzania: A cross-sectional hospital-based study. BMC Public Health. 2012;12(1):1.

24. Moshi FV, Millanzi WC, Mwampagatwa I. Factors Associated With Uptake of Iron Supplement During Pregnancy Among Women of Reproductive Age in Tanzania: an Analysis of Data From the 2015 to 2016 Tanzania Demographic and Health Survey and Malaria Indicators Survey. Front Public Heal. 2021;9(July):1–8.

25. Nimwesiga C, Murezi M, Taremwa IM. Adherence to Iron and Folic Acid Supplementation and Its Associated Factors among Pregnant Women Attending Antenatal Care at Bwindi Community Hospital, Western Uganda. Int J Reprod Med. 2021;2021:1–10.

26. Fite MB, Roba KT, Oljira L, Tura AK, Yadeta TA. Compliance with Iron and Folic Acid Supplementation (IFAS) and associated factors among pregnant women in Sub- Saharan Africa: A systematic review and metaanalysis. PLoS One [Internet]. 2021;16(4 April):1–17. Available from: http://dx.doi.org/10.1371/journal.pone.0249789

27. Wambugu J, Chege P, Muga M. Maternal determinants of non-compliance to folic acid supplementation and iron among pregnant women in Nakuru North Sub-County, Kenya. J Med Heal Sci. 2022;1(1):46–54.

28. Azanaw MM, Gebremariam AD, Dagnaw FT, Yisak H, Atikilt G, Minuye B, et al. Factors associated with numbers of antenatal care visits in rural ethiopia. J Multidiscip Healthc. 2021;14:1403–11.

29. Acup W, Opollo MS, Akullo BN, Musinguzi M, Kigongo E, Opio B, et al. Factors associated with first antenatal care (ANC) attendance within 12 weeks of pregnancy among women in Lira City, Northern Uganda: a facility-based cross-sectional study. BMJ Open. 2023;13(7):1–8.

30. Ba DM, Ssentongo P, Kjerulff KH, Na M, Liu G, Gao X, et al. Adherence to Iron Supplementation in 22 Sub-Saharan African Countries and Associated Factors among Pregnant Women: A Large Population-Based Study. Curr Dev Nutr. 2019;3(12):1–8.

31. Bancha B, Lajore BA, Petros L, Hassen H, Jemal A. Time to non-adherence to iron and folic acid supplementation and associated factors among pregnant women in Hosanna town, South Ethiopia: Cox-proportional hazard model. PLoS One [Internet]. 2022;17(9 September):1–13. Available from: http://dx.doi.org/10.1371/journal.pone.0275086

32. Park CY, Eicher-Miller HA. Iron Deficiency Is Associated with Food Insecurity inPregnant Females in the United States: National Health and Nutrition Examination Survey 1999-2010. J Acad Nutr Diet. 2014;114(12):1967–73.

Response to reviewers' matrix

Manuscript title: Iron and folic acid supplements uptake among pregnant women in Dar es Salaam, Tanzania

Reviewer #1

SN	Reviewer's comment	Authors' response	Page number
1	Is there specific medical reasons why women are sent to referral hospitals in this region? Are the pregnant individuals different from those that would normally visit non-referral hospitals? Curious if this would influence the generalizability of the results?	Thank you for this comment.<br>Some of the pregnant women from health primary care facilities are referred to tertiary facilities owing to either complications, in need of more specialized care, or for caesarean section delivery. Some, owing to history of complicated previous delivery, have their first ANC booking in these tertiary facilities, and others do register without any complication. However, there are no restrictions applied that limit women staying close to these facilities from receiving care. Owing to the diversity of these reasons, women treated in these hospitals would be more likely similar to those in other urban facilities. We have added this detail in the discussion section.	Page 14, line 279-280
2	Data in tables is presented oddly. Each percentage reported is for each line of the variable, which is mis-leading. Suggest for it to be switched so that percentages reflect each variable as grouped, as a total of 100% represented vertically (rather than horizontally).	Thank you. We have now changed the row percentages to be column percentages.	Page 10, Table 1
3	Also there are percentages and other data missing from some categories, and suggest to be consistent in either presenting whole numbers or numbers with decimal places for each percent reported.	Thank you for the observation.<br>We have reported all proportions in one decimal point. The odds ratios and 95% CI are in two decimal places, and all p-values in three decimal places.	All
4	Why not consider IFA uptake as a continuous number, to have more power to detect associations across the measured variables/predictors?	We thank the reviewer for this comment. We analysed IFAS uptake as a categorical variable for comparison with the recommended minimum intake by the WHO. While measuring IFAS uptake as a continuous variable would have increased our	Page 7, Line 159-164

		statistical power, we believe reporting the recommended minimum intake in terms of number of days that participants took the tablets would be better for use in clinical settings and policy implications especially in LMICs and with particular context of countries with high burden of anemia with a minimal qualified health workforce attending high number of clients.	
	Line 144: It is not clear how the RCH card was used to 'verify' the number of IFA tablets consumed. Was it just written in as a number by women or health care providers?	The RCH cards are filled by the health care providers during each ANC visit. The health care providers document the number of IFA tablets provided to the mothers among other information. The cards were used to verify if the women attended the ANC visits sufficiently to receive the IFA tablets as recommended and received the reported number of pills. We acknowledge that this may not necessarily mean they consumed the provided medications. We have reported this limitation in the discussion section.	Page 13, line 266-268
	Line 147: 200 mg ferrous sulphate should correspond to an exact content of elemental iron, not a range of 30-60 mg. Please check and correct.	Thank you. We have added the exact elemental iron content (65mg) that is equivalent to 200mg ferrous sulphate.	Page 7, line 157
	Line 153-4: "adequate IFA uptake is defined as four IFA tablets every week" – who defined this? Is there a reference that could be included here for context?	We thank the reviewer for this observation. We have revised this to include the standard recommended dose by WHO. The WHO recommends daily uptake of IFAS throughout pregnancy. We have considered a cutoff point of 90 or more pills as previously reported in similar settings and adopted one of the 2022 TDHS cut off points (1-3).	Page 7, Line163-165
	What about women with severe anemia – did they receive any other intervention or suggested dosing regime (e.g. supplements with a higher iron content)? Or IV infusions?	Yes, although we did not collect data on Anaemia in this study, severe anaemia may warrant admission and more investigation and management. As per the national guidelines, women with severe anaemia receive additional services including blood transfusions (those who were not in labour period), a higher supplement dosage of 120 mg of elemental iron and 400 µg folic acid. They are also counselled on dietary intake of higher iron and folic acid contents.	NA
	Line 99: why did you only include women with a prior history of ANC? How many women were excluded for these exact reasons? This	Thank you. In Tanzania, all women attending ANC visits are provided with IFAS for free and this is also recorded in their ANC cards. We only included women who attended at least one ANC to assess uptake of IFAS in a	Page 14, line 287-289

	would directly influence the generalizability of your results especially if you excluded the most vulnerable people with poor access (equity)	setting where IFAS is freely and equitably available. Women with no prior ANC attendance may have lacked that free access. We would also not be able to countercheck the self-report of IFAS uptake with ANC attendance and records. Of note, we would like to point out that according to the Tanzania Demographic Health Survey (TDHS), the coverage of a single ANC visit in Tanzania is high (more than 90%) and would not have significantly affected our estimates. We have added implication for future research to address this gap.	
	Acceptability is a very important consideration and factor as to why pregnant individuals may not be consuming the IFA. Was this assessed in the questionnaire?	Thank you for the question. That is correct, however, we did not assess acceptability as a co-variate in our study.	NA
	Line 53: thresholds for anemia during pregnancy actually have different trimester-specific thresholds. Please see the latest 2024 WHO guidance on thresholds for anemia in individuals and populations.	Thank you for the observation. We have added the correct definition according to trimesters. Anemia in pregnancy is diagnosed as hemoglobin concentration of less than 11.0 g/dl in the first trimester, 10.5g/dl in the second trimester and 11.0g/dl in the third trimester.	Page 3, line 53-54
	Line 55: “significantly dependent on iron” – iron is a component of the RBC, suggest to rephrase this text.	We have rephrased this sentence. Thank you.<br>Iron is a crucial component of hemoglobin, a protein found in red blood cells (RBCs) that is responsible for carrying oxygen throughout the body.	Page 3, line 56-57
	Line 66: “due to the lower bioavailability of nutrients in pregnant women” please add a reference for this statement.	We have added the appropriate reference reporting lower bioavailability of nutrients in pregnancy. Thank you.	Page 3, line 71
	Line 69: “… there is a considerable risk that iron deficiency and folic acid deficiency may manifest” – in fact, there are much more important consequences for both mother and infant that could be referenced here.	Thank you for the comment. We have added additional maternal consequences such as reduced physical capacity, infections, and postpartum depression. Infant consequences include low birth weight, neural tube defects, and preterm birth.	Page 3-4, line 77-81
	Line 118: TDHS – which one?	This was the recent 2022 TDHS report. We have added the year. Thanks.	Page 6, line 127-128
	Line 211: Explain the results reported in the Mbeya region (rather than just saying they were ‘similar’)	We thank the reviewer for this observation. This has been revised. We have made comparison with the 2022 Tanzania Demographic Health Survey which reported	Page 12, Line 220-222

		coverage of IFAS by number of days similar to our methodology (1).	
	Line 213: reference to Moshi study – did they assess IFA in the similar approach, considering 90 tablets as the threshold for adequate intake? Was it self reported?	In the Moshi study, the information on folic acid and iron was retrieved from the antenatal record and further information was obtained by interview with responses "Yes" or "No" to the questions "Did you take any drugs during this pregnancy?"<br>This study reported on any IFA intake and did not measure the adequacy of intake. We have added the details on IFA measurements used as part of what could experience the observed difference. Thank you.	Page 12, line 222-225

References

1 Mainland] MoHMT, [Zanzibar] MoHM, (NBS) NBoS, (OCGS) OotCGS, ICF. Tanzania Demographic and Health Survey and Malaria Indicator Survey (TDHS-MIS). Dodoma, Tanzania; 2022.

2 Lyoba WB, Mwakatoga JD, Festo C, Mrema J, Elisaria E. Adherence to Iron-Folic Acid Supplementation and Associated Factors among Pregnant Women in Kasulu Communities in North-Western Tanzania. International Journal of Reproductive Medicine. 2020;2020:1-11.

3 Nisar YB, Dibley MJ, Mir AM. Factors associated with non-use of antenatal iron and folic acid supplements among Pakistani women: a cross sectional household survey. BMC Pregnancy and Childbirth. 2014;14(1):305.
