# Supplementary material for: Uptake of iron and folic acid supplements among pregnant women in Dar es Salaam, Tanzania
Source: PLOS Glob Public Health. 2025 Oct 6;5(10):e0005278. doi: 10.1371/journal.pgph.0005278 (PMC12500133; doi:10.1371/journal.pgph.0005278)
Supplement: Attachment — Submitted filename: Response to reviewers matrix_24May2025.docx [file pgph.0005278.s003.docx]

**Response to reviewers’ matrix**

**Manuscript title: Iron and folic acid supplements uptake among pregnant women in Dar es Salaam, Tanzania**

**Reviewer #1**

| **SN** | **Reviewer’s comment** | **Authors’ response** | **Page number** |
| --- | --- | --- | --- |
| 1 | Is there specific medical reasons why women are sent to referral hospitals in this region? Are the pregnant individuals different from those that would normally visit non-referral hospitals? Curious if this would influence the generalizability of the results? | Thank you for this comment.  Some of the pregnant women from health primary care facilities are referred to tertiary facilities owing to either complications, in need of more specialized care, or for caesarean section delivery. Some, owing to history of complicated previous delivery, have their first ANC booking in these tertiary facilities, and others do register without any complication. However, there are no restrictions applied that limit women staying close to these facilities from receiving care. Owing to the diversity of these reasons, women treated in these hospitals would be more likely similar to those in other urban facilities. We have added this detail in the discussion section. | Page 14, line 279-280 |
| 2 | Data in tables is presented oddly. Each percentage reported is for each line of the variable, which is mis-leading. Suggest for it to be switched so that percentages reflect each variable as grouped, as a total of 100% represented vertically (rather than horizontally). | Thank you. We have now changed the row percentages to be column percentages. | Page 10, Table 1 |
| 3 | Also there are percentages and other data missing from some categories, and suggest to be consistent in either presenting whole numbers or numbers with decimal places for each percent reported. | Thank you for the observation.  We have reported all proportions in one decimal point. The odds ratios and 95% CI are in two decimal places, and all p-values in three decimal places. | All |
| 4 | Why not consider IFA uptake as a continuous number, to have more power to detect associations across the measured variables/predictors? | We thank the reviewer for this comment. We analysed IFAS uptake as a categorical variable for comparison with the recommended minimum intake by the WHO. While measuring IFAS uptake as a continuous variable would have increased our statistical power, we believe reporting the recommended minimum intake in terms of number of days that participants took the tablets would be better for use in clinical settings and policy implications especially in LMICs and with particular context of countries with high burden of anemia with a minimal qualified health workforce attending high number of clients. | Page 7, Line 159-164 |
|  | Line 144: It is not clear how the RCH card was used to ‘verify’ the number of IFA tablets consumed. Was it just written in as a number by women or health care providers? | The RCH cards are filled by the health care providers during each ANC visit. The health care providers document the number of IFA tablets provided to the mothers among other information. The cards were used to verify if the women attended the ANC visits sufficiently to receive the IFA tablets as recommended and received the reported number of pills. We acknowledge that this may not necessarily mean they consumed the provided medications. We have reported this limitation in the discussion section. | Page 13, line 266-268 |
|  | Line 147: 200 mg ferrous sulphate should correspond to an exact content of elemental iron, not a range of 30-60 mg. Please check and correct. | Thank you. We have added the exact elemental iron content (65mg) that is equivalent to 200mg ferrous sulphate. | Page 7, line 157 |
|  | Line 153-4: “adequate IFA uptake is defined as four IFA tablets every week” – who defined this? Is there a reference that could be included here for context? | We thank the reviewer for this observation. We have revised this to include the standard recommended dose by WHO. The WHO recommends daily uptake of IFAS throughout pregnancy. We have considered a cutoff point of 90 or more pills as previously reported in similar settings and adopted one of the 2022 TDHS cut off points (1-3). | Page 7, Line163-165 |
|  | What about women with severe anemia – did they receive any other intervention or suggested dosing regime (e.g. supplements with a higher iron content)? Or IV infusions? | Yes, although we did not collect data on Anaemia in this study, severe anaemia may warrant admission and more investigation and management. As per the national guidelines, women with severe anaemia receive additional services including blood transfusions (those who were not in labour period), a higher supplement dosage of 120 mg of elemental iron and 400 μg folic acid. They are also counselled on dietary intake of higher iron and folic acid contents. | NA |
|  | Line 99: why did you only include women with a prior history of ANC? How many women were excluded for these exact reasons? This would directly influence the generalizability of your results especially if you excluded the most vulnerable people with poor access (equity) | Thank you. In Tanzania, all women attending ANC visits are provided with IFAS for free and this is also recorded in their ANC cards. We only included women who attended at least one ANC to assess uptake of IFAS in a setting where IFAS is freely and equitably available. Women with no prior ANC attendance may have lacked that free access. We would also not be able to countercheck the self-report of IFAS uptake with ANC attendance and records. Of note, we would like to point out that according to the Tanzania Demographic Health Survey (TDHS), the coverage of a single ANC visit in Tanzania is high (more than 90%) and would not have significantly affected our estimates. We have added implication for future research to address this gap. | Page 14, line 287-289 |
|  | Acceptability is a very important consideration and factor as to why pregnant individuals may not be consuming the IFA. Was this assessed in the questionnaire? | Thank you for the question. That is correct, however, we did not assess acceptability as a co-variate in our study. | NA |
|  | Line 53: thresholds for anemia during pregnancy actually have different trimester-specific thresholds. Please see the latest 2024 WHO guidance on thresholds for anemia in individuals and populations. | Thank you for the observation. We have added the correct definition according to trimesters. Anemia in pregnancy is diagnosed as hemoglobin concentration of less than 11.0 g/dl in the first trimester, 10.5g/dl in the second trimester and 11.0g/dl in the third trimester. | Page 3, line 53-54 |
|  | Line 55: “significantly dependent on iron” – iron is a component of the RBC, suggest to rephrase this text. | We have rephrased this sentence. Thank you.  Iron is a crucial component of hemoglobin, a protein found in red blood cells (RBCs) that is responsible for carrying oxygen throughout the body. | Page 3, line 56-57 |
|  | Line 66: “due to the lower bioavailability of nutrients in pregnant women” please add a reference for this statement. | We have added the appropriate reference reporting lower bioavailability of nutrients in pregnancy. Thank you. | Page 3, line 71 |
|  | Line 69: “… there is a considerable risk that iron deficiency and folic acid deficiency may manifest” – in fact, there are much more important consequences for both mother and infant that could be referenced here. | Thank you for the comment. We have added additional maternal consequences such as reduced physical capacity, infections, and postpartum depression. Infant consequences include low birth weight, neural tube defects, and preterm birth. | Page 3-4, line 77-81 |
|  | Line 118: TDHS – which one? | This was the recent 2022 TDHS report. We have added the year. Thanks. | Page 6, line 127-128 |
|  | Line 211: Explain the results reported in the Mbeya region (rather than just saying they were ‘similar’) | We thank the reviewer for this observation. This has been revised. We have made comparison with the 2022 Tanzania Demographic Health Survey which reported coverage of IFAS by number of days similar to our methodology (1). | Page 12, Line 220-222 |
|  | Line 213: reference to Moshi study – did they assess IFA in the similar approach, considering 90 tablets as the threshold for adequate intake? Was it self reported? | In the Moshi study, the information on folic acid and iron was retrieved from the antenatal record and further information was obtained by interview with responses “Yes” or “No” to the questions “Did you take any drugs during this pregnancy?”  This study reported on any IFA intake and did not measure the adequacy of intake. We have added the details on IFA measurements used as part of what could experience the observed difference. Thank you. | Page 12, line 222-225 |

**References**

1	Mainland] MoHMT, [Zanzibar] MoHM, (NBS) NBoS, (OCGS) OotCGS, ICF. Tanzania Demographic and Health Survey and Malaria Indicator Survey (TDHS-MIS). Dodoma, Tanzania; 2022.

2	Lyoba WB, Mwakatoga JD, Festo C, Mrema J, Elisaria E. Adherence to Iron-Folic Acid Supplementation and Associated Factors among Pregnant Women in Kasulu Communities in North-Western Tanzania. International Journal of Reproductive Medicine. 2020;2020:1-11.

3	Nisar YB, Dibley MJ, Mir AM. Factors associated with non-use of antenatal iron and folic acid supplements among Pakistani women: a cross sectional household survey. BMC Pregnancy and Childbirth. 2014;14(1):305.
