# Supplementary material for: Uptake of iron and folic acid supplements among pregnant women in Dar es Salaam, Tanzania
Source: PLOS Glob Public Health. 2025 Oct 6;5(10):e0005278. doi: 10.1371/journal.pgph.0005278 (PMC12500133; doi:10.1371/journal.pgph.0005278)
Supplement: Attachment — Submitted filename: Response to reviewers matrix_25Aug2025.docx [file pgph.0005278.s005.docx]

**Response to reviewers’ matrix**

**Manuscript title: Uptake of iron and folic acid supplements among pregnant women in Dar es Salaam, Tanzania**

**Reviewer #1**

| Reviewer’s comment | Authors’ response | Page number |
| --- | --- | --- |
| Iron and folic acid supplements (IFAS)? | Thank you. We have added the word “supplements” | Page 2, Line 27 |
| increase instead of raise sounds better | We have replaced raise with “increase” | Page 3, Line 68 |
| cannot be met sounds better than cannot be satisfied | We have replaced “satisfied” replaced with “met” | Page 3, Line 69 |
| are these not causes of iron deficiency and not signs of the deficiency? Correction required. | These are maternal consequences of anaemia as reported in the references | Page 3, Line 76-81 |
| What about levels of iron? Do they influence infants' intellectual development? | Levels of iron also influence infants’ intellectual development. We have added “iron” to the statement. Thank you. | Page 3, Line 85-86 |
| supplements | Thank you. ‘s’ added to the word supplement. | Page 4, Line 92 |
| 'assess levels and determinants of IFAS uptake during pregnancy'  from the title, abstract, introduction to methodology the broad objective of the study should be aligned or should be consistent. | Thank you. We have aligned the broad objective in the title, abstract, introduction and methodology as suggested. | Page 5, Line 97-99 |
| Only women with a prior history of antenatal care visits to any health facility during their most recent pregnancy and who had their antenatal care (ANC) cards for quality checks were included.  Note a change of the line structure. | Thank you. We have revised this statement for clarity. | Page 5, Line 108-110 |
| achieved instead of required | We have replaced “required” with “achieved” as suggested | Page 5, Line 119 |
| added instead of used | We have replaced “used” with “added” as suggested | Page 5, Line 120 |
| written instead of prepared | We have replaced “prepared” with “written” as suggested | Page 6, Line 131 |
| Data on maternal age, education level, employment status, and  wealth index was collected.  Note the change of the line structure. | Thank you. We have rephrased the sentence as suggested | Page 6, Line 133-134 |
| which was used to determine economic status  Note addition of which | The word “which” added to the sentence | Page 6, Line 136 |
| to create is better than to produce | We have replaced “produce” with “create” as suggested | Page, 6Line 138 |
| HCP should be written in full in the first instance and then abbreviated subsequently. | We have replaced HCP with its long form (Healthcare professional) | Page 6, Line 141 |
| A roster of twelve foods was extracted from the 2022 Tanzania Demographic Health Survey (TDHS)  Note change of the line structure | The sentence has been revised as suggested | Page 6, Line 140-142 |
| Participants were asked to mention the food items they had consumed on the previous day, and  the final score for dietary diversity was determined.  Note change of the line structure. | The sentence has been revised as suggested | Page 6, Line 147-149 |
| previously referred to as respondents  Are the two synonymous? | We have maintained the use of “respondents” throughout | Page 7, Line 155 |
| Adequate IFAS uptake was defined as the intake of folic acid and iron tablets for a minimum of 90 days per pregnancy adopted from  previous studies in similar settings from Tanzania and Pakistan and using TDHS methodologies.  Note change of the line structure | The sentence has been revised as suggested | Page 7, Line 163-165 |
| IFAS uptake was categorized as normal uptake (90 or  160 more pills per pregnancy) and low uptake (less than 90 pills per pregnancy).  Note change of the line structure | The sentence has been restructured for clarity as suggested | Page 7, Line 172-174 |
| This line needs to be restructured for better communication.  The magnitude of IFAS uptake was calculated as the percentage of IFAS uptake of  169 90 pills or moreover the total study population. | We have rephrased IFAS uptake definition as follows:  IFAS uptake was defined as the proportion of respondents who reported IFAS intake of 90 pills or more during their recent pregnancy | Page 7, Line 175-176 |
| are they factors associated with low IFAS intake or are they determinants of IFAS intake?  As stated earlier, consistency is required throughout the write up | We have revised this as determinants of IFAS uptake throughout the manuscript | Page 10-11 |
| This line needs to be restructured for better communication.  The magnitude of IFAS uptake was calculated as the percentage of IFAS uptake of  90 pills or more over the total study population. | We have reconstructed the sentence for more clarity. It now reads  The magnitude of IFAS uptake was calculated as the percentage of IFAS uptake of  90 pills or more over the total study population. | Page 7, Line 174-176 |
| Check earlier comment on whether they are factors associated with low uptake? and now here is; factors associated with adequate uptake or they are determinants of IFAS uptake?  Consistency is required | We have revised this as determinants of IFAS uptake throughout the manuscript | Page 12, Line 201 |
| These IFAS uptake findings are also not very different from evidence from a neighboring country, Kenya  Note that this line has been restructured | We have reconstructed the sentence for more clarity. | Page 13, Line 229 |
| This line is not clear hence it requires restructuring | We have reconstructed the sentence for more clarity. It now reads:  “However, this study only reported on IFA supplements intake without reporting the adequacy of IFA intake.” | Page 13, Line 233-234 |
| This line is not clear. It should be rewritten to communicate | We have reconstructed the sentence for more clarity. It now reads:  “This highlights that the optimal IFAS uptake is significantly lower than the recommended optimal uptake, immediate action is therefore needed to address this gap.” | Line 229 |
| what does this mean? The line requires restructuring | We have restructured the sentence; it now reads as follows  The current findings show an improvement compared to a previous study conducted in Moshi Tanzania in 2009, where 16.1% of women reported taking folic acid and iron supplements | Page 12, Line228-229 |
| Did this study establish healthcare workers’ attitudes? If yes, how were the attitudes measured? This needs to come out in the methodology. | Thank you for this question.  We included a single question in the questionnaire to document overall health care workers’ attitude from the respondent’s perspective. We have added details in the methods section  Here is the question included in the questionnaire  What was the attitude of the health care worker towards you during ANC visits?   1. Good 2. Fair 3. Bad 4. Don’t know | Page 7, Line 142-144 |
| were these findings from this study? if so, then these statements should be written in the past tense. | We have revised these statements to past tense as follows  “In this study, the wealth index was also a determinant of IFAS uptake among pregnant women; those from wealthier households were more likely to have adequate uptake.” | Page 14, Line 264-267 |
| Replace our with this | We have replaced “our” with “this” | Line 284,285,286,304 |

**Reviewer #2**

| Line 25 of the abstract: suggest to revise 'biggest brunt' to 'highest prevalence observed' | Thank you. We have made the revision. It now reads  “Anemia in pregnancy is a public health concern globally with the highest prevalence observed in low and middle-income countries” | Page 2, Line 25-26 |
| --- | --- | --- |
| Suggest to use the acronym IFA rather than IFAS, especially as in the abstract the word 'supplement' is not included at first mention. IFA is more common. | We thank the reviewer for this comment.  It was recommended to use IFAS by the first reviewer – we have maintained the same acronym throughout the manuscript as suggested. | Page 2, Line 27 |
| Consider slight rewording of title to: "Uptake of IFA..." rather than IFA uptake..." | We have rephrased the title to “Uptake of iron and folic acid supplements among pregnant women in Dar es Salaam, Tanzania” | Page 1, Line 1 |
| Line 31 suggest rewording to 'women post-delivery' rather | We have reworded and restructured the sentence as suggested | Page 2, Line 34 |
| Line 35 -instead of "one in three" please report the actual observed n= from the study | Thank you  We have added the numbers as follows  Only 136 pregnant women (31.8%) who attended antenatal care (ANC) at referral hospitals had an appropriate IFAS uptake. | Page 2, Line 39 |
| Line 55 - anemia does not need to be capitalized | We have made the necessary changes. Thank you. | Page 2, Line 55 |
| Several acronyms are used throughout but not consistently. Check line 109 'ANC' and check line 93 'IFA' and also comprehensively check all acronyms with a search and replace to ensure consistency throughout. | We have edited and used consistent acronyms throughout the manuscript, especially IFAS and ANC as key acronyms. Thank you. | All pages |
| reference to the WHO recommendation should include a citation | Thank you. We have added the appropriate citation as suggested. | Line 233 |
| As this journal does not have a copy editor, strongly suggest that the grammar be reviewed before the final version is submitted. | Thank you. We have done a comprehensive review and language editing of the whole manuscript. | All pages |
